# Perceiving material qualities from moving contours

**DOI:** 10.1038/s41598-026-46015-w

**Published:** 2026-04-14

**Authors:** Amna Malik, Ying Yu, Huseyin Boyaci, Katja Doerschner

**Affiliations:** 1 Department of Psychology, Justus Liebig University Giessen, Otto-Behaghel-Strasse 10F, 35394 Giessen, Germany; 2 Departments of Psychology and Neuroscience, and A.S. Brain Research Center & National Magnetic Resonance Research Center (UMRAM), Bilkent University, Ankara, Turkey

**Keywords:** Dynamic line drawings, Dynamic dot materials, Contour motion, Material perception, Dynamic contours, Motion perception, Materials science, Psychology, Psychology

## Abstract

While research on the perception of line drawings has long demonstrated the importance of contours in object recognition, recent work shows that contours can also convey material properties. For example, even simple 2D shapes with varying contours have been shown to evoke vivid impressions of different materials. However, such static representations capture only a single moment in time. When a material moves, its contours shift, evolve, or deform over time, creating contour motion. Does this contour motion convey diagnostic information about material properties, independent of surface appearance? Existing studies on the role of dynamic cues in material perception either use fully rendered 3D stimuli, where contour motion is confounded with rich surface information, or motion-only displays (dynamic dot stimuli or noise patches), which eliminate surface cues but also lack clearly defined contours. As a result, the relative contribution of contour motion to material perception remains unclear. To address this gap, we measured how human observers perceive materials from dynamic line drawings (“line”), compared to animations of fully textured stimuli that carry optical and motion information (“full”), as well as dynamic dot stimuli (“dot”). Stimuli were three rendered versions (full, dot, line) of material animations from five material categories (jelly, liquid, smoke, fabric, and rigid-breakable). In one experiment, participants rated five material attributes (dense, flexible, wobbly, fluid, airy motion), and in a second experiment, participants were asked to choose one of the two materials that is more similar to a third material across all possible combinations. Results from both experiments consistently show that perceptual organization in both line and dot conditions strongly corresponds to that observed in the full condition. A control rating experiment with static line drawings (single images) showed significantly weaker correspondence to the full condition than between dynamic line drawings and the full condition, indicating that contour motion, not static shape alone, drives the effect. Together, these findings show that contour motion provides diagnostic information for material perception by jointly conveying contour shape and its time-varying dynamics, extending beyond what can be inferred from static contour cues alone.

**Supplementary Information:**

The online version contains supplementary material available at 10.1038/s41598-026-46015-w.

## Introduction

Lines play a fundamental role in visual perception. This is evident from the earliest known cave drawings, dating back at least 45,500 years, where early humans used lines to depict objects, animals, and scenes from life^1^. The persistence of line-based representation across human history suggests that our visual system is attuned to processing lines as an essential cue for recognizing shapes, forms, and structures in our environment. Research shows that humans can recognize objects and scenes from line drawings with remarkable efficiency, even in the absence of shading, texture, or color cues^2–7^. The question is, what makes line drawings so effective? To answer this, we should consider what lines represent. Line drawings primarily correspond to meaningful edges (contours) in real-world images. In the absence of surface texture, contours of an object can correspond to: occluding contours (such as smooth occlusions, where the surface of the object curves away from the viewer, crease occlusions, where surfaces meet at a sharp edge), surface creases (folds or ridges that indicate internal shape structure), or illumination-related contours (like shadows and specular highlights). While all of these can be depicted in line drawings, studies have shown that occluding contours and creases are especially informative for recognizing object shape and identity^8,9^.

Since object recognition from line drawings is very robust, it has inspired edge-based theories of object recognition, such as those proposed by Biederman^10^. According to these accounts, contours provide the critical structural information needed for identification, while surface properties such as texture or color play a minimal role. Material perception, in contrast, has placed comparatively less emphasis on the role of contours as surface cues appear to be central for estimating and recognizing material properties^11^. With few exceptions, the role of contours in material perception has received little attention. Pinna and Deiana^12^ provided compelling evidence that contours alone provide substantial information about material properties. In one of the experiments, they presented 2D squares depicted with various contour shapes and asked observers to identify the material. Participants spontaneously attributed distinct material identities to each square based on the shape of the contour alone, e.g., straight edges were seen as plastic, wavy contours as fabric, and sharp spikes as metal. In another experiment, they showed multiple squares to participants with each square retaining a clean outline except for one locally distorted edge^12^. These subtle distortions, jagged notches, rounded bulges, or irregular outflows, led observers to infer events like tearing, melting, or spilling. Remarkably, these static line drawings elicited vivid impressions of what the object was made of and what had happened to it based on contour variation^13–15^.

**Fig. 1:**
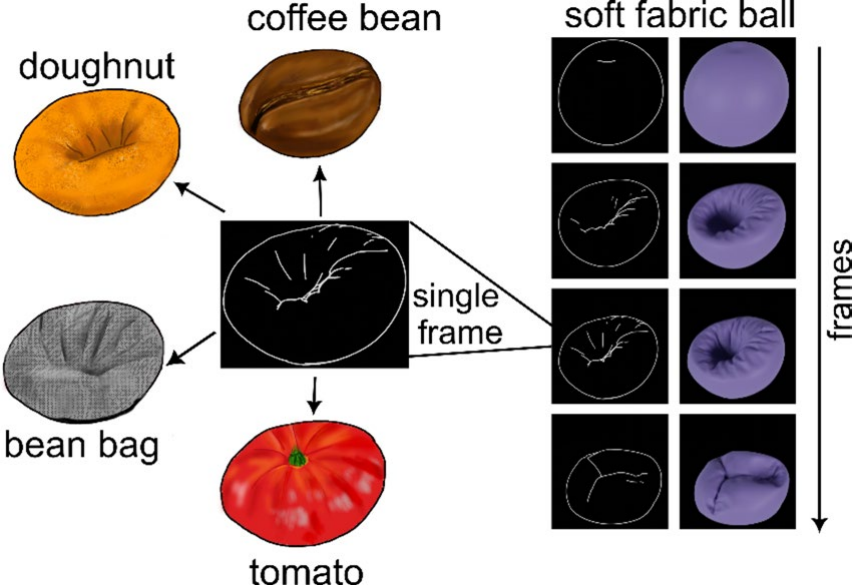
A static frame extracted from an animation depicting a soft fabric can be interpreted ambiguously as different objects. However, when viewed dynamically across multiple frames, contour motion makes the material identity immediately apparent.

However, it’s important to consider that real-world materials are seldom static; instead, they frequently change as they interact with external forces. While some, such as glass or brittle objects, break upon impact, others, like rubber or jelly, temporarily deform before returning to their original shape when interacted with. Moreover, inherently dynamic materials like liquids and smoke continuously change shape, even in the absence of direct external forces. As a result, contours evolve over time, reflecting characteristic changes unique to each material, and these changes may provide an even richer source of information, capturing aspects of material behavior that static line drawings cannot convey. For example, consider Fig. 1(left panel), which shows one of the frames extracted from an animation. This image can be interpreted in multiple ways: it might resemble a doughnut, a bean bag, a coffee bean, or a tomato. However, when the stimulus is viewed dynamically across multiple frames, the material identity becomes immediately clear as the contours move (right panel of Fig. 1), namely a balloon, made of a very soft fabric.

Here, we tested whether two cues emerging from contour motion, shape changes over time, and motion cues such as speed, acceleration, etc., can explain how humans perceive material qualities from line drawings. Previous research showed that both shape and motion cues play a role in the perception of material qualities^16–22^. However, these studies either used fully rendered 3D stimuli, where contour motion is confounded with rich surface information, or motion-only displays (dynamic dot stimuli or noise patches), which eliminate surface cues but also lack clearly defined contours. As a result, the specific contribution of contour motion to material perception remains unclear. One notable exception is Kawabe & Nishida^19^, who isolated contour motion (only smooth occlusions) by removing specular and diffuse reflections from a jelly cube’s surface. Their findings showed that elasticity judgment was influenced by contour deformation, but their study was limited to a single material (jelly) and a single property (elasticity). Here, we aimed to investigate the role of contour motion across a broader range of materials using both semantic and non-semantic tasks.

**Fig. 2:**
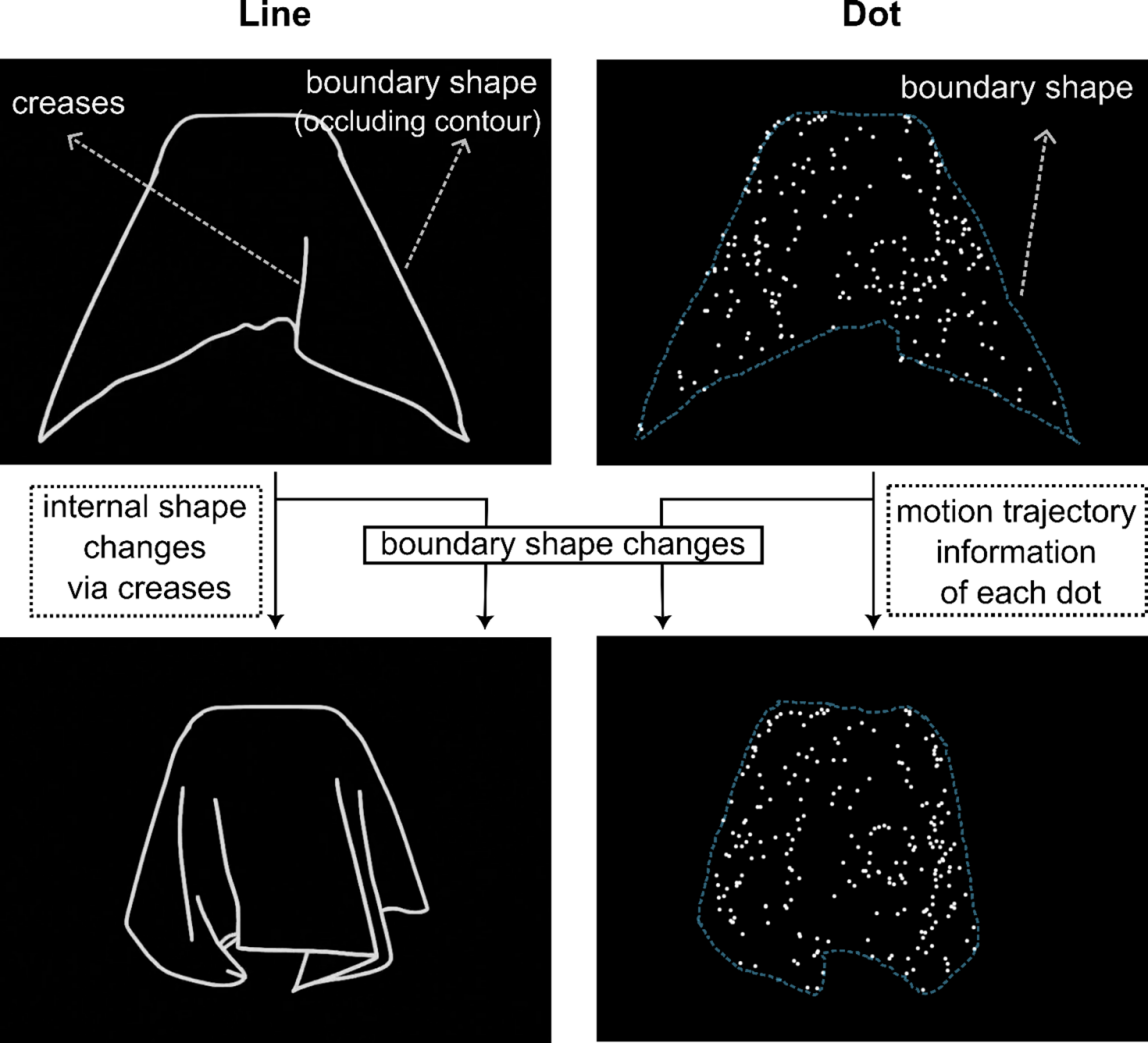
Representative frames from one of the animations are shown for both the line and dot conditions. Line drawings depict occluding contour motion (conveying boundary shape changes) and crease/internal contour motion (showing how contours evolve within the object’s boundary). Dot stimuli convey internal motion cues through the trajectories of individual dots and provide coarse structural information from their instantaneous positions. The blue dotted line in the dot condition is included for illustrative purposes to highlight how boundary shape changes can also be inferred from the dot stimuli, although this cue is weaker than in the line condition.

To isolate contour motion, we used dynamic line drawings (line drawing animations), rendering only contours (both occluding contours and creases) of an object. We also rendered a dynamic dot version of stimuli as used by Schmid & Doerschner^21^ and Bi et al.^17^. Dynamic dot stimuli depict material motion by dots stuck in or on the surface of the material, thereby removing optical cues. They primarily provide internal motion cues, where each dot changes position over time, generating apparent motion signals through its trajectory. The instantaneous positions of dots at any given moment define the object’s overall structure. Temporal integration of this structural information enables perception of how the object’s 3D shape and surface evolve over time. To some extent, dynamic dot stimuli also convey changes in occluding contour, although much weaker than line-based representations (see Fig. 2). In contrast, dynamic line drawings make boundary shape changes explicit by directly representing occluding contour motion (OCM). In addition, they also provide information about how contours evolve over time within the object’s boundary (internal creases), referred to as internal contour motion (ICM). Together, these characteristics of line drawings make them a reliable source of contour motion. Here, we investigated the relative contribution of contour motion in material perception using these dynamic line drawings. Specifically, we asked whether contour motion conveyed by line drawings leads to material perception similar to that evoked by full-textured stimuli, and if so, how does it compare to dot stimuli?

**Fig. 3:**
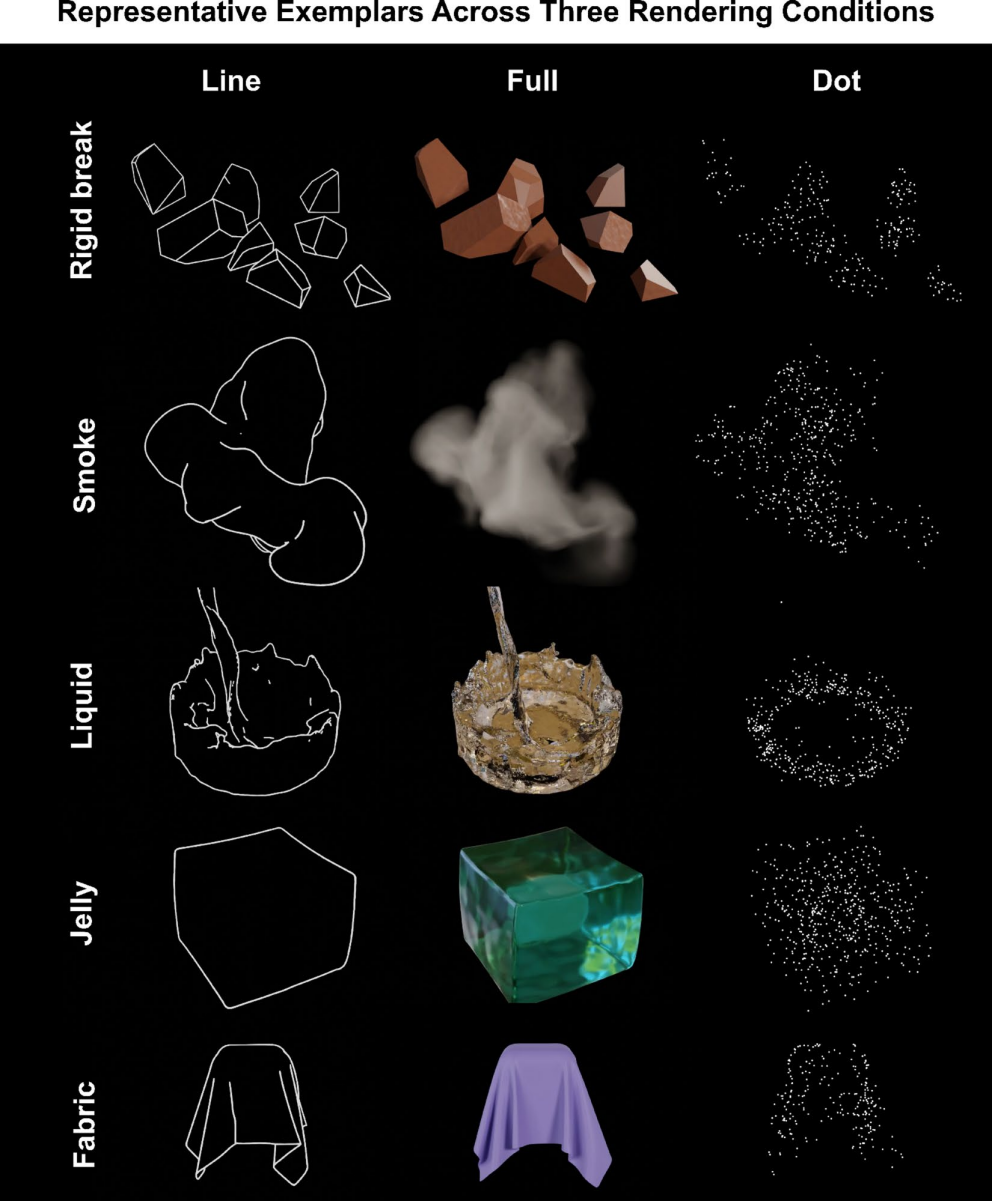
Rendering conditions for stimuli: Each column represents one of the three rendered versions of stimuli, showing a single frame from a representative exemplar for each material category.

To test this, we created computer-generated animations representing five material categories: jelly, liquid, smoke, fabric, and rigid-breakable, as shown in Fig. 3. These animations featured randomly shaped objects reacting to external forces, exhibiting their dynamic behaviors such as deformation, motion, or dispersion. We rendered each animation in three versions: line, full, and dot. We conducted two experiments: a rating experiment using a semantic task in which participants rated five material attributes (dense, flexible, wobbly, fluid, airy motion), and a similarity matching experiment using a non-semantic task, in which participants viewed animations of materials in a triplet-alternative forced-choice setting and were instructed to choose one of the two materials that was more similar to the third material across all possible combinations. The latter experiment served to validate whether the chosen attributes sufficiently capture the perceptual space. Results from both experiments suggested that contour motion plays a critical role in the perception of mechanical material qualities. An additional control rating experiment with static line drawings (single images) showed significantly weaker correspondence to the full condition than between dynamic line drawings and full condition, indicating that contour motion, not static shape alone, drives the effect. Finally, in order to explore what information our visual system extracts from these dynamic stimuli, we estimated several motion and shape statistics for line and dot stimuli and correlated them with the perceptual data. We found that the estimated motion or shape-derived statistics can only partially explain perceptual differences across material categories. This suggests that more work is needed to discover and quantify visual features that drive the perception of material qualities in dynamic scenes.

## Materials and methods

### Stimuli

To create a stimulus set with a sufficiently broad range of material classes, we rendered animations of materials from five distinct categories: jelly, liquid, smoke, fabric, and rigid-breakable. Each category contained multiple animations representing different variations within the same material class, which we refer to as *exemplars*. Specifically, the jelly, fabric, and rigid-breakable categories each included four exemplars, while the liquid and smoke categories each contained three exemplars. Figure S1 shows representative frames (frames 3, 15, and 40) from exemplars of each material category. All animations were rendered from six or eight camera angles, yielding 114 animations in total. Each animation comprised 48 frames, presented at a rate of 24 frames per second, resulting in a 2-second video. We rendered the animations at a resolution of 1024 × 1024 pixels. In all animations, the ground was set to invisible, and objects were presented against a solid black background. We wanted to ensure that participants do not form vivid impressions or associations of exemplars with any specific object to minimize the likelihood of recalling properties solely based on the object presented in a single frame. Therefore, we deliberately kept the shapes of the objects as generic as possible, such as cubes and spheres, to prevent them from resembling any specific or recognizable objects based on their geometry.

We used Blender version 3.3.0^23^, an open-source 3D computer graphics software tool, to create animations for our study. We utilized Blender’s different simulation systems to achieve realistic effects across various materials and their interactions with external forces. Details about the rendering of each exemplar from all categories can be found in *Appendix A*. We rendered three versions of each animation, as described next.

### Rendering conditions

1. Line: To create a line drawing version of animations, we used the grease pencil toolbox within Blender, enabling us to render only occluding contours and creases. A grease pencil object was created, and a line art modifier was added to trace the contours of the selected object. Most settings remained at default, with the line color set to white. Line thickness was adjusted as a function of distance from the camera, maintaining a ratio of line thickness to camera distance between 2 and 3. This ensured uniform thickness despite different camera specifications across exemplars. The final animations rendered only the Grease Pencil outlines (occluding contours and creases), without materials or background, using Blender’s Cycles render engine. Representative frames for one exemplar from each category are shown in the left column of Fig. 3.
2. Dot: To generate dynamic dot versions of animations, we imported 3D vertices of each material’s mesh from Blender into MATLAB (version 2022a, MathWorks Inc., New York, NY, USA) and computed 2D projections of vertices based on the camera angles used to render animations. We sampled a subset of 2D vertices and plotted a white dot on a black background for each vertex, frame by frame, to render an animation representing the motion of material through the dots. The number of sampled vertices varied across exemplars based on the area spanned by the material across frames. We first converted the fully textured animations to binary format and calculated the area spanned in each frame as the ratio of pixels with intensity 1 to those with intensity 0. To determine the number of sampled vertices, we used the 75th percentile of the distribution of the area spanned in each frame and multiplied it by 2000. The 75th percentile was chosen instead of the maximum area to avoid outliers, as the distribution of the area spanned across frames is not always normal. Some videos exhibit highly skewed distributions, where using the maximum area would lead to extreme values (See Supplementary Figure S2). By taking the 75th percentile, we ensured a relatively more stable and representative measure. Representative frames for one of the exemplars from each category are shown in the right column of Fig. 3, along with corresponding frames from the full condition in the middle column.
3. Full: Full-textured versions of animations (shown in Supplementary Figure S1 and middle columns of Fig. 3) were rendered with all cues, including optical, shape, and motion cues, using Blender’s Cycles render engine. In the remainder of this article, we will refer to it as the *‘full’* condition.

### Experiment 1: rating mechanical properties

In the first experiment, participants rated each stimulus on five mechanical material attributes. The experiment followed a between-subjects design with three rendering conditions: line, dot, and full, and 20 different observers participated in each condition. This design was used to prevent participants in the line or dot conditions from recognizing materials by referencing the full condition.

#### Participants

Sixty participants (12 male and 48 female, age range: 19–52 years; mean age: 26.25 years) participated in the experiment. All participants had normal or corrected-to-normal vision (self-report). Participants gave their written informed consent before the experiments. Experimental procedures were approved by the ethics board at Justus-Liebig-University Giessen and were carried out in accordance with the guidelines set forth by the Declaration of Helsinki. Participants were compensated at the rate of 8 euros per hour.

#### Attributes

**Fig. 4:**
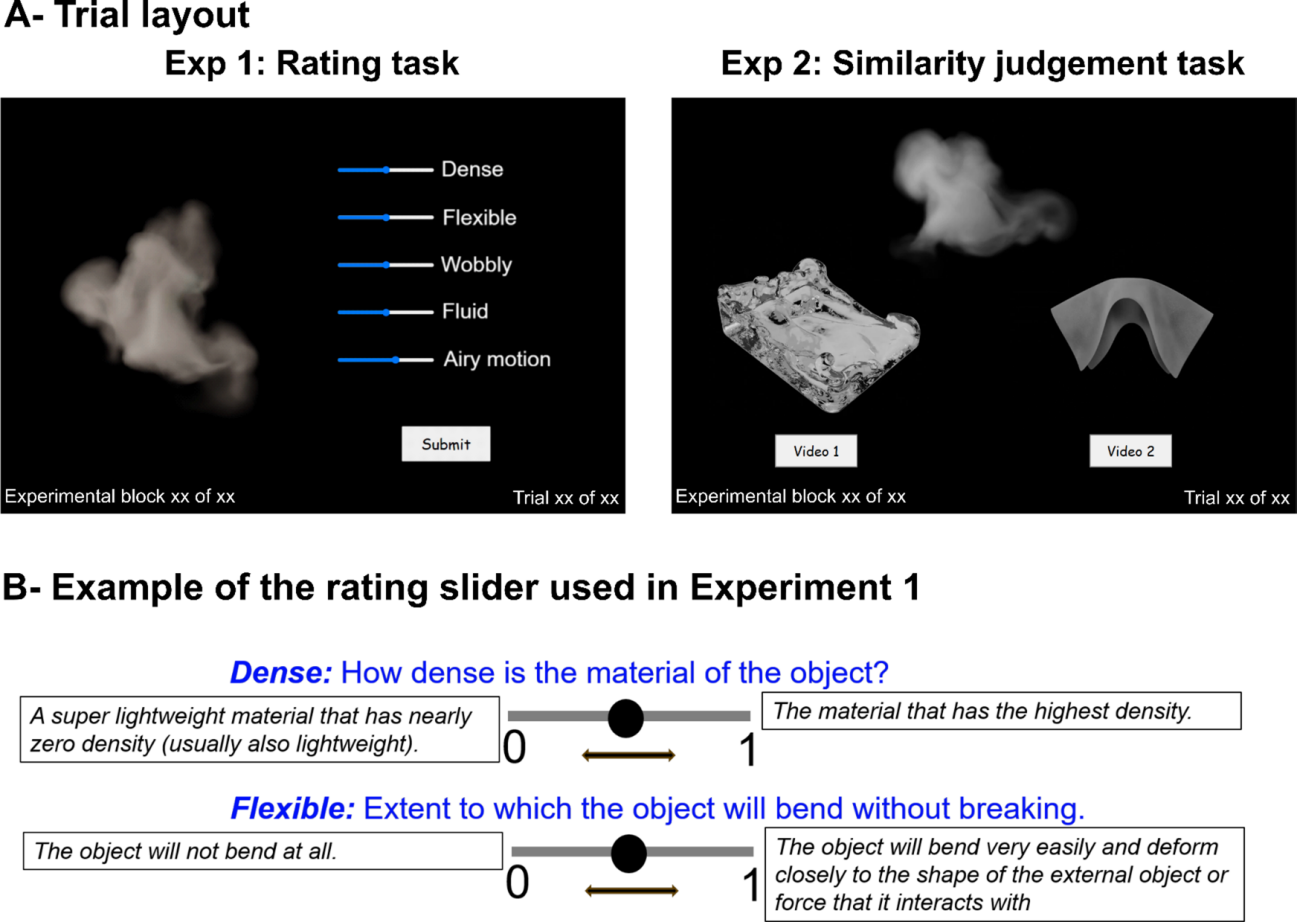
(**A**) An example of trial layout from Experiment 1 – Rating task (left panel), and Experiment 2 – Similarity judgment task (right panel). The text and images have been rescaled in the figure for clarity and do not reflect the actual scale used in the experiment. (**B**) Example of the rating slider used in Experiment 1, with criteria for scores 0 (left) and 1 (right) provided separately in participant handouts.

We selected five attributes (dense, flexible, wobbly, fluid, and airy motion) to characterize the mechanical qualities of the five material categories. Participants rated each attribute on a continuous scale from 0 to 1. Before the experiment, handouts were provided to ensure clarity, outlining each attribute and specifying the criteria for scores *0* and *1* (Fig. 4-A). A translated handout was available for German speakers, but the experiment was always presented in English. The following definitions in the handouts explained the five attributes and their ratings:

Dense How dense is the material of the object?

0: A super lightweight material that has nearly zero density (usually also lightweight).

1: The material that has the highest density.

Flexible The extent to which the object will bend without breaking.

0: The object will not bend at all.

1: The object will bend very easily and deform closely to the shape of the external object or force that it interacts with.

Wobbly The extent to which the object will shake or jiggle unsteadily while remaining intact.

0: The object will not wobble at all.

1: The object will wobble nonstop when triggered by a very subtle force.

Fluid The extent to which the object will flow.

0: The object will not flow at all.

1: The object will flow very easily and quickly.

Airy motion The extent to which the material floats and/or spreads in the air in a lightweight fashion.

0: The motion is not airy at all.

1: The motion is extremely airy.

#### Experimental setup

Stimuli were presented on a 27-inch LCD monitor (ViewSonic VA270-H, Walnut, California, USA) with a refresh rate of 100 Hz and a resolution of 1920 × 1080, controlled by a Dell system running on Windows 10 Pro. The experiment was developed using a combination of HTML and JavaScript and hosted on a local server. We used the JavaScript library p5.js to present our stimuli. Stimuli were presented as 24 frames/s videos; although the monitor refreshed at 100 Hz, the temporal sampling of the animations was determined by the video frame timing. The viewing distance was fixed at 70 cm. The experiment took place in a dark room.

#### Procedure

Upon arrival, participants signed the consent form and received detailed instructions outlining the task and attributes as described above. The experiment started with a welcome screen, and the subject ID was recorded. By pressing the ‘Enter’ button, they proceed to the experimental trials. On each trial, participants viewed the animation of material on the left side of the display, projecting a viewing angle of 18.8^°^. Five sliders corresponding to five material attributes were displayed on the right side of the display with a ‘submit’ button at the bottom (see left panel of Fig. 4-B). Participants were instructed to rate each attribute on a scale from 0 (left) to 1 (right) by dragging the slider with a mouse. The duration of each animation was 2 s (24 frames/s), which was replayed continuously until the participants completed rating five attributes and clicked the submit button to proceed to the next trial. The subsequent trial started with an interval of 300 ms. The position of the sliders is reset to the middle of the bar at the start of each trial. There was no time limit, but participants were encouraged not to spend more than 10 s on each trial. On average, they spent 20.4 s on one trial. The experiment consisted of 114 trials, with one animation per trial. Each unique stimulus was presented only once without repetition, and the order of presentation was randomized. The trial and block numbers were displayed on the bottom right and left corners, respectively.

#### Data analysis

All analyses and plotting were conducted in R (version 4.3.2) using RStudio (version 2023.9.1.494)^24^.

##### Inter-observer correlations

To assess the consistency of attribute ratings across participants, we computed inter-observer correlations. The ratings for all 114 movies (5 attributes per movie) from a single participant were flattened into a vector. The Spearman correlation between these vectors was estimated for each pair of participants, providing a single correlation coefficient per participant pair.

##### Factor analysis and clustering

 To compare perceptual organization across rendering conditions, we first reduced the correlated attribute ratings using exploratory factor analysis (EFA) and then applied Ward clustering in the resulting factor space to assess how strongly materials cluster by category, and whether this structure is preserved across rendering conditions. EFA was performed at the participant level (maximum-likelihood extraction with varimax rotation). Based on the eigenvalue criterion, we retained a two-factor solution and computed regression-based factor scores for each exemplar. We then summarized the structure in the 2D factor space using hierarchical agglomerative clustering with Ward’s method (Ward.D2) applied to Euclidean distances between exemplars’ mean factor scores. The number of clusters was fixed at k = 5 to enable direct comparison with the five predefined material categories.

##### Representational similarity analysis

To compare perceptual organization across tasks and rendering conditions, we used representational similarity analysis (RSA) to construct and correlate representational dissimilarity matrices (RDMs). To obtain rating RDMs, we calculated a ‘1 - Spearman’ correlation between the ratings for each pair of exemplars across all participants. Correspondence between RDMs from different rendering conditions was assessed using the Mantel test. For each comparison, Mantel’s r, indicating Spearman’s correlation between the RDMs, was computed, along with a p-value (adjusted for multiple comparisons) based on 999 permutations to assess statistical significance.

### Experiment 2: similarity judgement task

In the second experiment, we used a similarity judgment task in a triplet two-alternative forced-choice (2-AFC) setting to measure the perceived dissimilarities between materials using the same set of stimuli as in Experiment 1 (Rating task), without requiring participants to rely on semantic labels. The only difference was that we used black-and-white animations for the full condition to avoid color as a strong cue for grouping materials together. The experiment followed a between-subjects design, with three rendering conditions: line, dot, and full, each presented to a separate group of participants.

#### Participants

We recruited 100 participants (no exclusions; 32 male, 67 female, one other; age range: 19–38 years; mean age: 26.25 years). Before the experiment, participants gave written informed consent. Experimental procedures were approved by the ethics board at Justus-Liebig-University Giessen and were carried out in accordance with the guidelines set forth by the Declaration of Helsinki. Participants were compensated at the rate of 8 euros per hour.

#### Experimental setup

This experiment was conducted online, hosted on Amazon S3, and developed using HTML and JavaScript. We used the JavaScript library p5.js to present our stimuli^25^. Participants completed the experiment using a web browser on their personal computers, with the recommended browser being Google Chrome. We instructed participants not to use mobile phones or tablets for the experiment. We offered participants the option to complete the experiment in either English or German, allowing them to choose their preferred language.

#### Procedure

Upon accessing the website, the experiment started with a *welcome screen* that provided general instructions about the online experiment and also directed participants to the informed consent. Next, participants completed a screen calibration procedure (based on the method developed by Li et al.^26^; see Appendix B for details). After calibrating their screens, participants filled out a *demographic form*, providing only age and gender information. They were then provided with information about the experiment and task on an *instruction screen* before the experiment began. They entered the experiment by clicking an ‘Enter’ button at the bottom of the instruction screen. On each trial, participants viewed the three animations, each belonging to a different material category. Animations were displayed in a triangular setting, as shown in the right panel of Fig. 4-B. The size of the animations was adjusted according to viewing distance such that each animation projected a viewing angle of 10^°^. The animation presented at the top was designated as the *reference* stimulus, while the two shown at the bottom served as *test* stimuli. Participants were asked to choose which of the two test animations was more similar to the reference animation, based on the perceived material. They indicated their choice by clicking the button located below the selected animation. The buttons were outlined in red from the beginning of the trial until the videos played once, i.e., for 2000 ms. We instructed participants not to respond until the outline disappeared. Even if they tried to respond before that, the subsequent trial would not start, forcing them to view the animations at least once. There was no time limit, but they were encouraged not to spend more than 10 s on each trial. On average, they spent 5.6 s on one trial. After they responded, the subsequent trial started with an interval of 300 ms. We also included catch trials to ensure participants maintained their focus throughout the experiment. If a participant consistently chose the same-sided video for more than 14 consecutive trials, or if their reaction times (measured from the time after the red line from the response buttons disappeared) were consistently shorter than 350ms, they were presented with a catch trial. During catch trials, participants were shown a warning message: ‘Are you paying attention?’ followed by an unrelated task (e.g., counting the number of circles among different shapes) to re-engage their attention. In the collected data, this criterion was never met; therefore, no trials were excluded on this basis.

#### Construction of representational dissimilarity matrices (RDMs)

To derive the perceived similarity between material categories, we followed the approach of Schmidt et al.^27^, where RDMs are constructed from participants’ similarity judgments in a triplet-based task. These judgments are aggregated across all triplet contexts to compute pairwise similarity scores, which are then transformed into dissimilarity values (see Appendix C for full details).

Correspondence between rendering conditions was assessed by comparing RDMs across different rendering conditions within the similarity judgment task. Additionally, to assess cross-task correspondence, RDMs from the similarity judgment task were compared with those from the rating task using the same statistical tests as described in Experiment 1.

#### Control experiment: Rating task with static line drawings

Because the dynamic line drawings may preserve strong static shape cues that could account for the similarity structure even without motion, we ran a static control experiment to test the contribution of contour shape alone. For each animation, we extracted a single frame (frame 24, the middle frame) from the corresponding stimulus from the dynamic line drawing animation and presented it as a static image. A separate set of 20 naive observers (3 male, 16 female, one other; age range: 20–48 years; mean age: 27.8 years) rated the same five attributes using the identical slider interface and instructions as in the main rating task. Analyses (EFA, Ward clustering, and RSA) were carried out using the same pipeline as for the dynamic conditions. Differences in correspondence to the full-condition RDM (Spearman Mantel r) between static line, dynamic line, and dynamic dot conditions were assessed using permutation tests, with Holm correction for multiple comparisons.

#### Motion and shape statistics

To identify features critical for estimating material properties, we analyzed motion and shape statistics derived from full-textured animations. We computed 20 motion statistics following the methodology of Kawabe et al.^18^ and 20 shape statistics following the methodology of Paulun et al.^28^. Motion statistics comprised four statistical moments, mean, standard deviation (SD), skewness, and kurtosis, computed for five distinct image-based motion features: image speed, divergence, curl, gradient, and discrete Laplacian. These calculations were implemented in MATLAB using vertices projected onto 2D frames (refer to Kawabe et al.^18^ for details). We computed a set of 20 shape-related features capturing aspects such as curvature, orientation, spatial distribution, and overall geometry of the objects in the animation frames (refer to Paulun et al.^28^ for details). Shape computations utilized binary masks obtained from the projected 2D animation frames and were likewise implemented in MATLAB. For each of the 114 animations, each shape feature was represented by a 48-dimensional vector (one value per frame), whereas each motion feature was represented by a 47-dimensional vector (one value per frame difference).

The analysis was done separately on shape and motion statistics. First, each feature column was transformed into z-scores so that all features contribute equally to downstream analyses. We then performed principal component analysis (PCA), retaining the minimal number of components that explained at least 80% of the variance. We then aggregated the resulting PC scores by computing the mean score of each retained component across all 48 frames of each video, yielding one representative PC score vector per video. These representative vectors defined each video’s coordinates in the PCA space. Because the motion and shape statistics are computed from 2D projections, they can vary with camera angle. Therefore, for the primary analysis, we randomly selected one camera angle per exemplar to avoid mixing heterogeneous camera angle dependent statistics. Importantly, although viewpoints occupied different regions of PCA space, different views of the same exemplar remained locally clustered (see Supplementary Figure S5). To further assess whether our conclusions depend on the particular viewpoint sampled, we repeated the complete analysis across many randomly selected camera angles (1000 seeds) and evaluated the stability of regression and correlation outcomes across seeds.

To evaluate how well motion and shape statistics predicted each of the five perceptual outcomes, we derived predicted attribute scores from the PCA feature space. Specifically, for each attribute (dense, flexible, wobbly, fluid, airy), we identified the reference animation with the lowest mean rating in Experiment 1 and computed the Euclidean distance from this reference to every other animation in the retained PCA space. Importantly, these predicted attributes are feature-derived proxy variables intended to quantify how much variance in perceptual judgments can be accounted for by the extracted statistics; they are not interpreted as a perceptual metric of attribute magnitude. We then conducted linear regression analyses relating each perceptual attribute rating (from Experiment 1) to its corresponding attribute predictor. Adjusted R-squared values were extracted to assess model fit, and p-values were computed to determine statistical significance. To maintain reliable statistical conclusions, we employed the Benjamini-Hochberg procedure for multiple comparison corrections, thereby controlling the false discovery rate.

In order to assess how well motion and shape statistics account for similarity judgements in Experiment 2, we constructed dissimilarity matrices based on the predicted attributes using the same methodology applied to the perceptual data. These RDMs were compared to the perceptual RDMs derived from the rating and similarity judgment tasks using the Mantel test. For each comparison, Mantel’s *r*, reflecting the Spearman correlation between RDMs, was computed alongside permutation-based p-values (adjusted for multiple comparisons), using 999 permutations to assess significance. All analyses and visualizations were performed in R (version 4.3.2) using RStudio (version 2023.9.1.494)^29^.

## Results

### Experiment 1: rating task

**Fig. 5:**
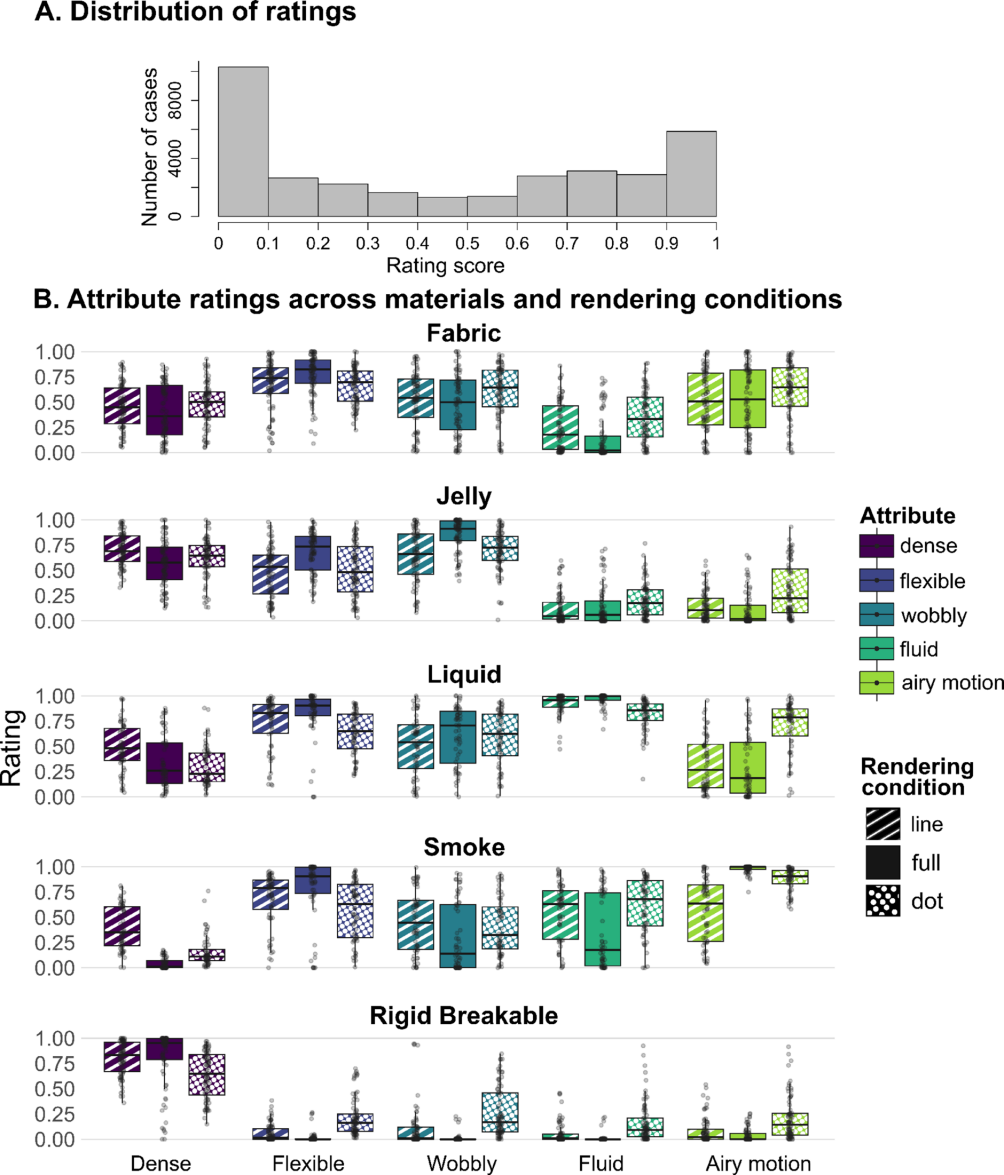
(**A**) Distribution of attribute rating values pooled across all participants, attributes, material categories, and conditions. (**B**) Box plots showing the distribution of ratings for each attribute across materials in the three rendering conditions (line, full, dot). Overlaid points indicate individual observations. Conditions are ordered as line, full, and dot.

The purpose of Experiment 1 was to assess whether participants are able to perceive mechanical qualities of materials based on contour motion alone (line drawing condition), and to compare their performance to that obtained with dynamic dot and full-textured animations. Participants viewed animations of materials and rated five mechanical attributes: density, flexible, wobbly, fluid, and airy motion, which were chosen to capture the characteristic features of the material categories used in our study. One of the common issues associated with the use of rating scales is the central tendency bias, where participants tend to avoid the extreme ends of the scale and prefer responses closer to the midpoint of the scale, leading to regression to the mean^30,31^. This bias can distort data by underrepresenting extreme values of responses. Given this concern, we first examined the distribution of our rating data. Figure 5-A shows the rating histogram pooling values across all participants, attributes, material categories, and conditions. This shows a bimodal distribution with clustering of responses to extreme values (0 and 1) rather than intermediate values. The frequent use of extreme values suggests that participants have strong categorical impressions of the perceptual qualities being evaluated. Thus, central tendency bias does not appear to be present in our data.

We also noted that the peak at the lower extreme is notably larger. While this asymmetry could suggest a potential response bias toward low values, it is more plausibly explained by the stimuli’s inherent characteristics. All the attributes in the rigid-breakable category except ‘dense’ naturally cluster around lower extreme values, contributing to this imbalance, as can be seen in the ratings of the rigid-breakable category in Fig. 5-B. Each material displays a unique characteristic pattern, with ratings spanning a range of values from high through intermediate to low, thus reflecting the distinct perceptual properties associated with each category.

#### Inter-observer correlations

We examined inter-observer correlations to determine whether participants were consistent in their ratings. If there is inconsistency, it suggests that the selected attributes are interpreted subjectively, potentially lacking clarity or shared meaning. Conversely, if participants are consistent in their ratings, it suggests that the attributes are meaningful and objective.

Supplementary Figure S3 shows the Spearman correlation between participants for all three rendering conditions. The mean inter-observer correlations for the line, full, and dot conditions are 0.75, 0.82, and 0.66, respectively. All inter-observer correlations were substantially positive for all conditions, ranging from 0.43 to 0.93, and were significant at the level of *p* < 0.001. The mean inter-observer correlation for the line and dot conditions was lower than for the full condition. However, considering that these were significantly reduced conditions of fully textured animations, the correlations remain high. Overall, the high inter-observer correlations for all conditions indicate that the selected attributes were meaningful and objective, effectively capturing the characteristic material properties across different categories. Another observation was that the inter-observer correlations were higher for the line condition compared to the dot condition, which indicates that the absence of clear contour cues leads to more ambiguity in inferring the mechanical attributes.

#### Factor analysis and clustering

Since the five attributes being rated were correlated with each other, we used factor analysis to reduce the attribute space. Note that our goal was not to determine the intrinsic low-dimensional structure of material perception per se. Rather, we used factor analysis as a descriptive dimensionality reduction step to summarize the correlated attribute ratings and enable a direct comparison of perceptual organization across the three rendering conditions (line, full, and dot) within a common representational space.

**Fig. 6:**
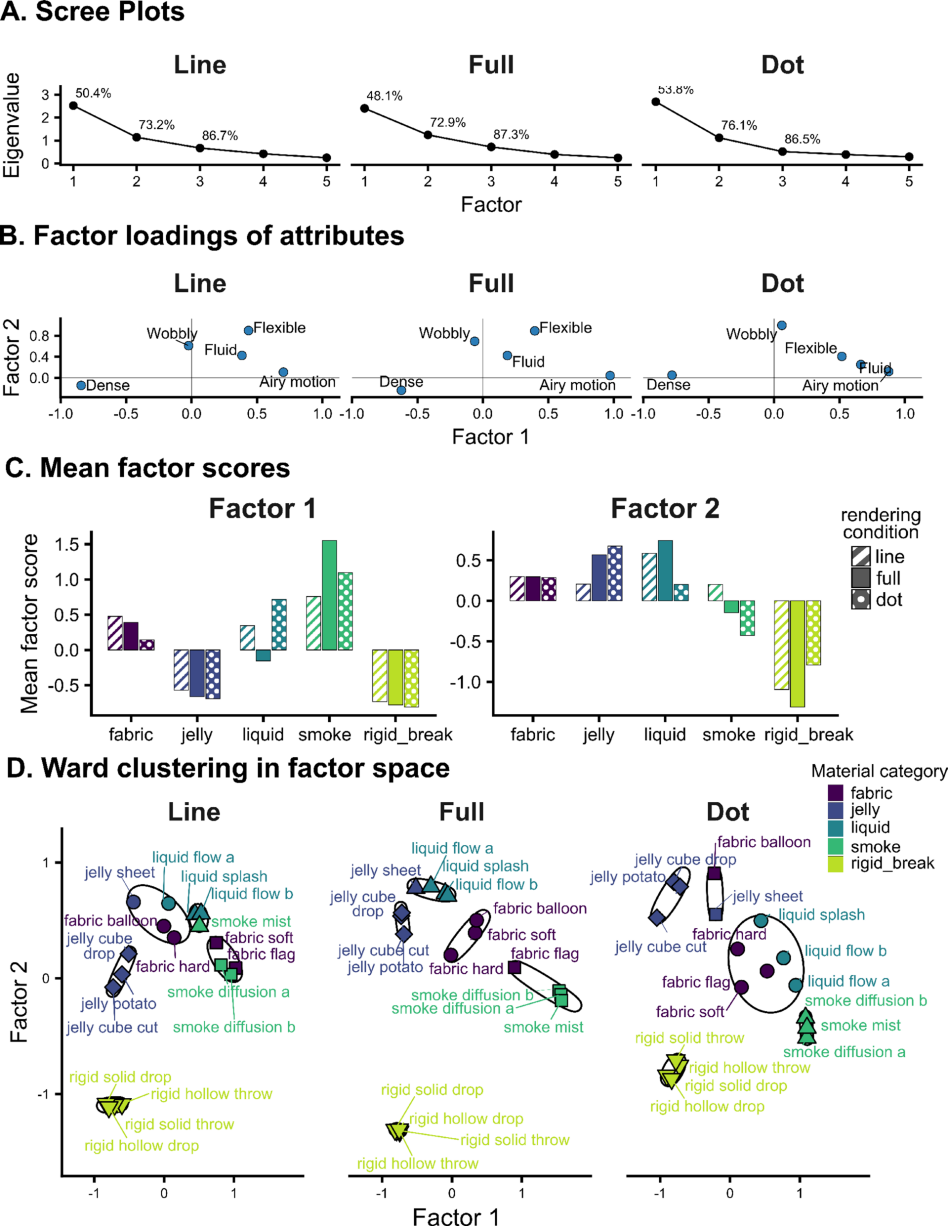
Factor analysis and Ward clustering of attribute ratings across rendering conditions. (**A**) Scree plots showing variance explained by the factors in the full, line, and dot conditions. (**B**) Attribute loadings for the two-factor solution in each condition (Factor 1: dense versus airy; Factor 2: deformability/compliance driven primarily by flexible and wobbly). (**C**) Mean factor scores for each exemplar in the two-dimensional factor space for each condition. (**D**) Ward hierarchical clustering based on Euclidean distances in the 2D factor-score space, cut at k = 5. Different colors denote material categories, and different shapes denote cluster membership.

The eigenvalue scree plots showed the first two principal components accounted for 72.9% of variance in full condition, 73.2% in line condition, and 76.1% in dot condition, as shown in Fig. 6-A. Because the incremental variance captured beyond Factor 2 was comparatively small across all conditions, we retained a two-factor solution for subsequent analyses.

Figure 6-B shows the attribute loading patterns for the three rendering conditions, highlighting that the two-factor loading structure was highly consistent across conditions. Factor 1 consistently contrasted *dense* (strong negative loading) with *airy motion* (strong positive loading), indicating a continuum from dense/compact to airy/light materials. Factor 2 was driven primarily by *flexible* and *wobbly* (and, to a lesser extent, *fluid*), consistent with a deformability/compliance dimension. Figure 6-C shows the factor scores (i.e., positions in factor space) for each material category in each condition. On Factor 1 (left panel), the ordering is largely preserved across conditions: smoke and jelly remain on the positive (airy) side, whereas jelly and rigid-breakable remain toward the negative (denser) side; liquid is the main exception, shifting to the positive side in the line and dot conditions. On Factor 2 (right panel), jelly and liquid tend to score higher while rigid-breakable score lower, with fabric and smoke showing relatively weak separation; however, separation along this axis is slightly compressed in the reduced conditions (e.g., jelly is lower in the line condition and liquid is lower in the dot condition), suggesting a slight shift in the underlying dimension in the absence of optical cues.

Figure 6-D visualizes Ward hierarchical clustering (Ward.D2) applied to Euclidean distances in the 2D factor score space. In the full condition, clustering reflects the strongest category like organization: exemplars separate cleanly along the two perceptual axes, with rigid-breakable grouping in the low deformability region (low Factor 2) and smoke grouping on the airy end of Factor 1. The remaining non-rigid materials occupy adjacent but distinguishable regions, yielding relatively coherent clusters. In the line condition, this organization is largely retained, but the spacing between non-rigid clusters is slightly reduced, producing more mixed boundaries. In the dot condition, clustering shows the greatest overlap among non-rigid categories, even though the overall factor-space organization remains similar.

Together, the consistent loading patterns and the largely preserved arrangement of exemplars across full, line, and dot conditions point to the same underlying perceptual dimensions, suggesting that contour motion and internal motion cues preserve key perceptual characteristics of materials.

#### Representational similarity analysis

**Fig. 7:**
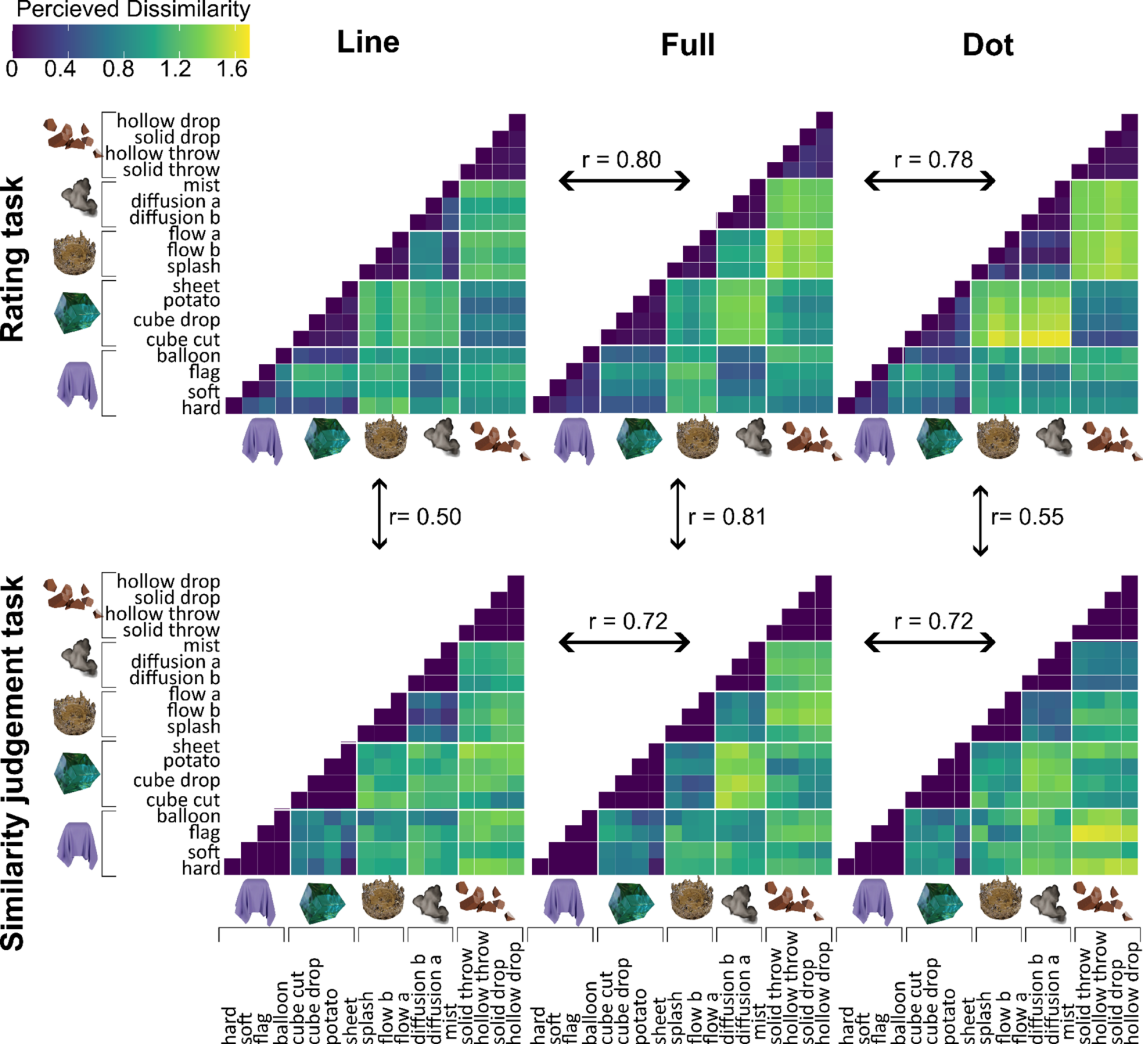
Representational Dissimilarity Matrices (RDMs) illustrating perceived similarities between material categories across three rendering conditions—line, full, and dot. The upper row displays RDMs derived from the rating task, while the lower row shows RDMs from the similarity judgment task. Arrows indicate the Mantel test correlations (r) between corresponding RDMs across tasks and rendering conditions.

RDMs across three rendering conditions: line, full, and dot, are shown in the upper row of Fig. 7, depicting the perceived differences between five material categories. Upon visual inspection of the RDM from the full condition (upper middle panel of Fig. 7), a clear pattern of perceptual differences emerges. Materials belonging to the same category were perceived as highly similar to each other while being more dissimilar to materials from other categories. Furthermore, the perceptual differences between categories appear consistent across exemplars within those categories. For instance, the rigid-breakable category is perceived as highly dissimilar to the liquid category, and this dissimilarity pattern holds consistently for all exemplars of liquid and rigid-breakable materials. This shows that the mechanical attributes we chose are characteristic properties of the material category and did not vary within the category, despite varied motion patterns within a category.

Next, to see whether these perceptual similarities are preserved when optical cues are removed, we compared the RDMs obtained from the line and dot conditions with the full condition. After correcting for multiple comparisons (*p* < 0.007), the Mantel test showed a strong and statistically significant relationship. Specifically, the Mantel test statistic ($\:r$) for the comparison between the full and line RDMs was *r* = 0.802, *p* = 0.001, and for the comparison between the full and dot RDMs, it was *r* = 0.784, *p* = 0.001. This correspondence is also visually evident in the patterns observed within the RDMs. However, some differences between line and dot conditions were apparent, which may offer insights into the relative importance of internal motion and contour motion cues in the perception of these material categories. Overall, the results strongly suggest that both line and dot conditions effectively convey the mechanical properties of materials.

### Experiment 2: similarity judgment task

To validate whether the five-dimensional space in Experiment 1 sufficiently characterizes the set of materials, and to ensure that perceptual dissimilarity measures were not biased by the use of a verbal task, we conducted an additional experiment. In this experiment, we constructed representational dissimilarity matrices (RDMs) based on non-verbal similarity judgments obtained through a triplet 2-alternative forced-choice (2-AFC) task. The lower row in Fig. 7 shows the RDMs derived from this experiment.

Visual inspection of the RDMs from the full condition of experiments 1 and 2 revealed consistent patterns between the rating and similarity tasks (see the top middle columns of Fig. 7 compared to the lower middle column). This correspondence is supported by very high r statistics derived from the Mental tests (*r* = 0.805, *p* = 0.001). Additionally, the correlation between the RDMs of line and dot conditions across task types is also significant (dot rating RDM and dot similarity judgment RDM: *r* = 0.555, *p* = 0.001; line rating RDM and line similarity judgment RDM: *r* = 0.497, *p* = 0.001), albeit lower than that observed for the full condition. These results suggest that the rating attributes we chose captured the perceptual space of materials fairly well and that it is not biased by having to make verbal responses.

Further, results indicate a statistically significant correlation between RDMs from line, dot, and full conditions (lower panel of Fig. 7). The Mantel test statistic ($\:r$) comparing the line and full RDMs is *r* = 0.721, *p* = 0.001, while the comparison between dot and full condition yielded *r* = 0.718, *p* = 0.001. These results align with the results from experiment 1, indicating that participants perceive materials in the line and dot conditions similarly to the full condition. The RDMs derived from the similarity judgment task revealed slightly greater differences between reduced (line and dot) and full conditions compared to those from the rating task. This suggests that when grouping materials by visual similarity, participants consider additional dimensions not captured in our rating experiment. We discuss this further below.

#### Control experiment: rating task with static line drawings

Results from the static line condition are shown in Supplementary Figure S4. The scree plot showed a dominant first factor (61.2% variance), with two factors explaining 75.1%, indicating a stronger reliance on a single dimension than in the dynamic conditions (Supplementary Figure S4-A). Loadings were also less differentiated: dense loaded in the opposite direction from the other attributes, but airy motion, fluid, flexible, and wobbly all loaded positively together (Supplementary Figure S4-B). Consistent with this, Ward clustering showed weaker category-like separation than in the dynamic conditions. Most notably, jelly and rigid-breakable categories collapsed into a shared region/cluster, and liquid exemplars split across clusters (Supplementary Figure S4-C). RSA results shown in Supplementary Figure S4-D also converged with these findings: the static line RDM corresponded less with the full RDM (Mantel’s *r* = 0.641, *p* = 0.001) than the dynamic line RDM (Mantel’s *r* = 0.803, *p* = 0.001), and this difference was significant (Δr = 0.162, Holm-corrected *p* < 0.05). Correspondence with the full condition did not differ between dynamic line and dynamic dot (Δr = − 0.018, *p* = 0.695). Overall, these results show that the static line condition preserves coarse grouping, but dynamic line drawings more strongly match the perceptual organization observed in the full condition.

#### Motion and shape statistics

**Fig. 8:**
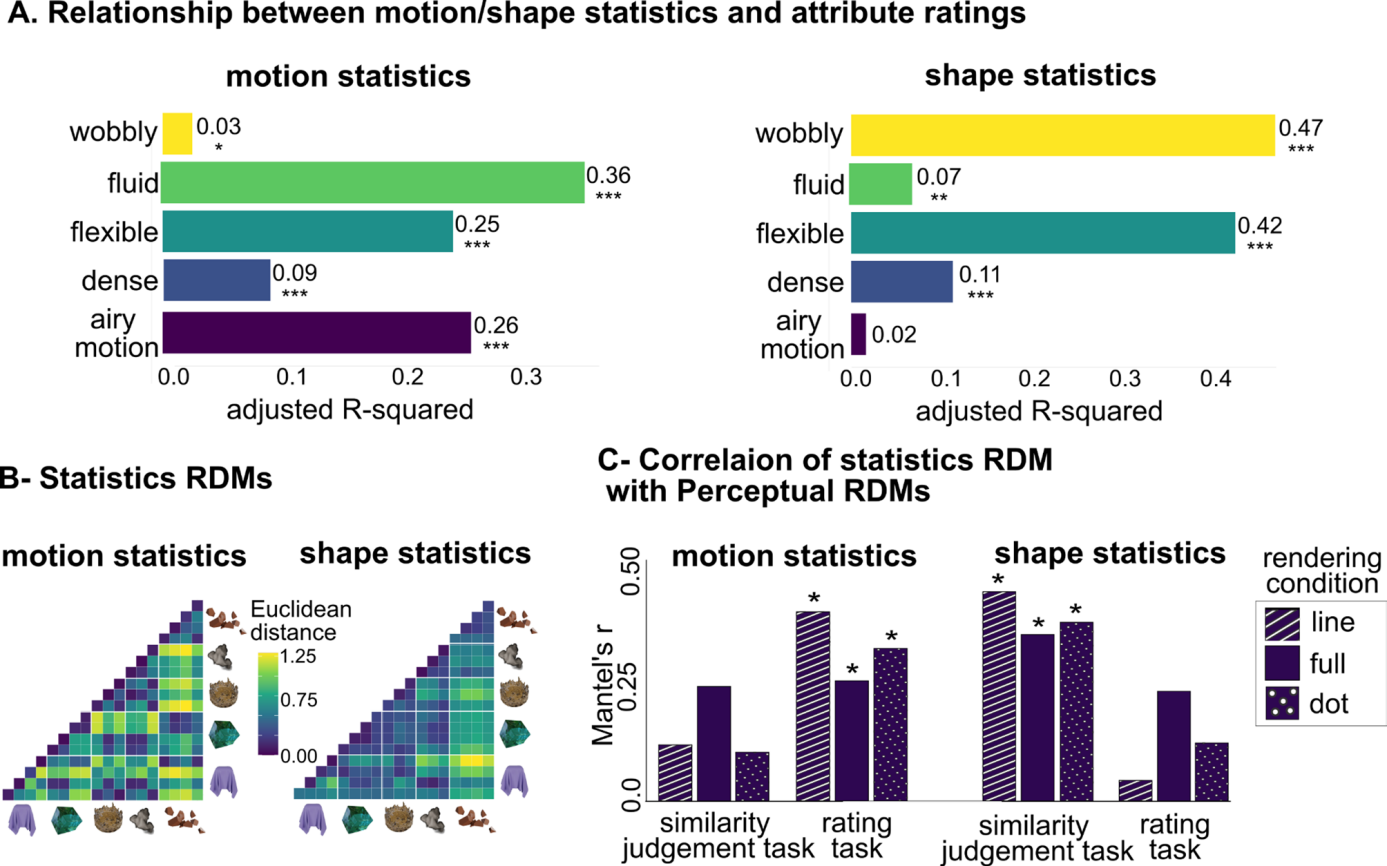
Relationship between perceptual attributes and underlying computational representations based on motion and shape features. (A) Adjusted R2 values from regression analyses predicting perceptual attributes with motion and shape statistics (* for *p* < 0.05, ** for *p* < 0.01, and *** for *p* < 0.001, corrected for multiple comparisons). (B) RDMs derived from motion and shape statistics. (C) Mantel’s r values comparing statistics RDMs to perceptual RDMs (rating and similarity judgments) across the line, full, and dot conditions (* for *p* < 0.05, corrected for multiple comparisons).

Figure 8-A shows the results of the regression analysis relating 20 motion and 20 shape features (described in the analysis), each to the 5 rated material attributes. Results from motion statistics suggest that *fluid* had the highest explanatory power (adjusted *R*^*2*^ = 0.36), followed by *airy motion* (adjusted *R*^*2*^ = 0.26), *flexible* (adjusted *R*^*2*^ = 0.25), *dense* (adjusted *R*^*2*^ = 0.09), and *wobbly* (adjusted *R*^*2*^ = 0.03). In contrast, shape-derived statistics demonstrated stronger predictive power for *wobbly* (adjusted *R*^*2*^ = 0.47) and *flexible* (adjusted *R*^*2*^ = 0.42), with lower values for *dense* (adjusted *R*^*2*^ = 0.11), *fluid* (adjusted *R*^*2*^ = 0.07), and *airy motion* (adjusted *R*^*2*^ = 0.02). These findings suggest that motion statistics and shape statistics are each predictive of different material attributes, and neither of them appears to be informative for predicting perceived density. We next conducted Mantel tests to compare RDMs derived from motion features with perceptual RDMs obtained through rating tasks and similarity judgments (Fig. 8). For the rating RDMs, we observed small-to-moderate correlations (Mantel’s *r* = 0.256–0.403) that remained significant after multiple-comparison corrections (*p*_*corr*_ = 0.012–0.036). Notably, the line condition showed the highest correlation among the rating RDMs. By contrast, correlations with the similarity judgement RDMs were small (Mantel’s *r* = 0.104–0.244) and were not significant. These findings indicate that motion-derived features capture participants’ attribute rating judgments more effectively than their similarity judgments.

In contrast, shape-derived RDMs exhibited the opposite pattern. For the rating RDMs, correlations were small (Mantel’s *r* = 0.043–0.232) and were not significant. However, correlations with the similarity judgment RDMs were moderate (Mantel’s *r* = 0.353–0.443) and reached significance in all conditions (*p*_*corr*_ = 0.006), with the line condition again yielding the highest correlation. These findings indicate that shape statistics capture participants’ similarity-based judgments more effectively than their attribute rating judgments.

To assess whether our findings were driven by camera angle differences, we reran the complete analysis repeatedly while randomly selecting a single camera angle per exemplar (1000 seeds). Across these repetitions, the regression and correlation outcomes showed the same overall pattern, indicating that our conclusions do not hinge on any particular camera angle choice (Supplementary Fig. 6).

## Discussion

Using animated line drawings, the current study investigated the relative contribution of contour motion in material perception. We compared perceptual judgments across three versions of rendered animations, line (depicting contour motion), full (with fully textured material), and dot (depicting dot motion), in two experiments: a rating experiment and a similarity judgment experiment. Across both tasks, perceptual organization in the line and dot conditions showed strong correspondence with the full condition, suggesting that contour motion in dynamic line drawings provides a powerful cue for material perception.

It has long been known that the information preserved in line drawing images serves as a basis for the visual recognition of objects and scenes^2–5,7,32^. However, it is not yet fully understood why line drawings are so robust. A possible explanation for the effectiveness of line drawings, as suggested by Sayim & Cavanagh^33^, is that they activate neural mechanisms originally adapted for processing natural environments. The primary visual cortex contains neurons specifically attuned to contour orientation, which recognize edges in natural settings rather than uniform regions. Due to their sensitivity to contrast and orientation patterns, these neurons not only detect real-world edges but also respond to drawn lines. Since lines in drawings are generally drawn at the locations where edges would appear in reality, these drawings activate the same perceptual processes as real-world images. Certain edges, such as occluding contours and creases, serve as key shape-defining features that allow the brain to infer the object’s underlying 3D structure from a 2D representation^9,34^. Hence, lines in line drawings serve as contours that convey the structure of 2D shapes, allowing the brain to infer the underlying 3D form. Moreover, neurons in V1 also detect the local motion of edges, while motion-sensitive areas such as MT integrate these signals to infer global object motion and transformations (Born & Bradley, 2005). Thus, the brain interprets moving lines in dynamic line drawings not as isolated features, but as edges of objects undergoing motion or shape changes.

Thus, our findings suggest that dynamic information in line drawings helps observers perceive not only shape transformations, such as how a fabric folds or stretches, but also motion characteristics, such as the rhythmic flapping of a flag, the speed of a falling rigid object, or the speed of flow of a liquid. Together, these cues allow the visual system to infer material properties efficiently. While prior research has provided important insights into how specific dynamic cues predict perceived material properties within a given category, for example, the relationship between flow speed and perceived viscosity of liquids^18^, between several motion statistics and perceived stiffness of fabrics^17^, or between deformation magnitude and perceived stiffness of jelly^20^, these studies have typically focused on single material classes. Materials from different categories can sometimes share similar dynamic properties. For instance, a fabric and a liquid might exhibit similar motion speeds when analyzed using optical flow, yet they differ so much in perceived viscosity. Likewise, a loosely woven cloth and a soft piece of jelly may undergo similar magnitudes of deformation, yet they are perceived to differ markedly in stiffness. So, how does the visual system identify whether an object is a piece of cloth, a stream of liquid, or a piece of jelly in the first place? Can observers distinguish between materials from different categories based on dynamic information alone? And if so, what specific image features does the visual system rely on to make these judgments? Our results suggest that contour motion conveyed solely through dynamic line drawings provides sufficient information for perception properties across a range of material categories, including jelly, liquid, smoke, fabric, and rigid-breakable materials.

While the current study highlights the importance of contour motion for material perception, it does not imply that motion is the only informative cue used by observers. Dynamic line drawings are informative precisely because they combine two sources of information: the static contour specifies shape, while motion specifies how that shape evolves through deformation and displacement over time. To dissociate the contribution of contour motion from static contour cues, we conducted a control experiment in which participants rated single frames extracted from the dynamic line drawing animations without observing any contour motion. Notably, results showed a substantial correspondence with the perceptual organization observed in the full condition (*r* = 0.64), indicating that static contour alone carries meaningful information about material. However, dynamic line drawings show markedly stronger correspondence (*r* = 0.80) compared to static line drawings, demonstrating that contour motion provides additional diagnostic information beyond what can be inferred from a single snapshot. This pattern suggests that material perception relies on complementary contributions from shape and motion cues in line with previous studies^19–21,35–37^.

### Perceptual consistency across ratings and similarity judgments

The rating task produced RDMs that closely aligned with those from the similarity judgment task, yielding a remarkably high correlation of 0.81 for the full condition. This result highlights two important implications. First, it has been argued that rating tasks rely on an observer’s ability to use language to describe material properties^38^. To address this potential linguistic bias, Experiment 2 employed similarity judgment, which bypassed explicit verbal encoding and provided a more direct perceptual assessment. Notably, while the rating experiment required participants to infer five material properties based on motion, shape, or optical cues, the similarity judgment task offered a purely non-verbal measure of how materials were grouped based on perceived properties. The strong agreement between these two tasks suggests that both verbal (semantic) and non-verbal (visual) judgments are interpreted through a shared underlying perceptual framework. This conclusion aligns with previous research showing a high degree of correspondence between the semantic representations of material categories and the attribute ratings of individual materials^39^ and between visual and haptic judgments of material properties^40^. While these studies have primarily provided indirect evidence of a link between verbal (semantic) and perceptual representations, our study offers a direct comparison between the perceptual space derived from verbal attribute ratings and non-verbal visual similarity judgments. Second, despite being limited to only five mechanical attributes, the rating task produced RDMs that are strongly correlated with similarity judgment RDMs. This is in line with findings of Schmid & Doerschner^21^, who demonstrated through factor analyses of material ratings that adjectives cluster around a few core dimensions rather than forming an exhaustive or independent set. They identified key perceptual dimensions such as hydration, fluidness of motion, airiness/density, hardness/softness, and smoothness. The attributes we selected (density, fluidity, flexibility, wobbliness, and airy motion) correspond closely to these dimensions. These findings are further supported by recent large-scale, data-driven work on the characterization of material representations^27^, showing that only a set of 36 dimensions can account for the majority of explained variance in human similarity judgments across materials sampled from 200 categories. Together﻿, these findings suggest that material categorization and similarity are governed by a common, low-dimensional perceptual structure that integrates both semantic and sensory information. Even when observers rate only a subset of mechanical material attributes, the resulting perceptual space is still highly predictive of general visual similarity judgments.

### Contribution of dynamic and optical cues to material perception

Despite high correspondence in the perceptual organization of line and dot condition with full condition, closer inspection reveals small yet meaningful deviations in how materials are perceived when optical cues are absent (line and dot conditions). These subtle deviations emphasize the complementary role optical cues play in the perception of material properties. For instance, in the factor space, liquids shift toward the airy end of factor 1 in the line and dot conditions (Fig. 6C, left), suggesting that when characteristic optical properties are absent, liquid is interpreted as less dense/compact and becomes perceptually closer to materials such as smoke. Consistent with this, RSA reveals increased similarity between liquid and smoke in line and dot conditions relative to the full condition (Fig. 7). In addition, clustering in the 2D factor-score space shows greater overlap among non-rigid categories in the line and dot conditions (Fig. 6D), indicating that category boundaries become less distinct as optical information is reduced. Together, these results suggest that motion cues conveyed through lines and dots preserve essential information required for material perception, but optical cues complement dynamic motion information and are essential for accurately distinguishing certain material categories.

Importantly, deviations from full condition are more pronounced in similarity judgment tasks than rating tasks, as indicated by slightly lower correlations between full and line conditions (0.72 similarity judgment vs. 0.80 rating) and between full and dot conditions (0.72 similarity judgment vs. 0.78 rating). We also observe notable qualitative changes in the patterns of similarity judgment RDMs when comparing different rendering conditions. For example, rigid breakable materials are perceived as more similar to smoke rather than fabric or jelly in the line and dot conditions, whereas under full conditions, these materials are perceived as more similar to fabric and jelly. Why are these shifts specific to the similarity judgment task and absent in the rating task? We suggest that similarity judgments rely on the interplay of both optical and motion cues to group materials meaningfully. Hence, when optical cues are missing (in line and dot condition), it leads to noticeable shifts in perceived similarity. In contrast, explicit attribute ratings of mechanical attributes (e.g., density or fluidity) rely predominantly on distinct dynamic motion cues that sufficiently convey specific properties independently. These findings are in line with prior research in liquid perception, where shape and motion cues dominate when participants rate liquid viscosity, but optical cues become more influential in a category naming task^22^. The category naming task, like our similarity judgment task, directly taps into underlying perceptual space. These results suggest that while dynamic cues play a critical role, they are not sufficient on their own; optical cues are also crucial, especially for capturing dimensions beyond those considered in our rating experiment.

This interpretation is further supported by notably weaker correlations between rating and similarity judgment RDMs in the line (0.50) and dot (0.55) conditions. These lower correlations arise because the removal of optical cues affects the two tasks differently: similarity judgments engage a broader set of perceptual dimensions that are more sensitive to the interplay of available cues, whereas attribute ratings focus on a distinct, limited set of dimensions that can often still be judged accurately using motion cues alone. As a result, when optical information is removed or unavailable, the perceptual space underlying similarity judgments is more substantially altered than that of attribute ratings. This task-specific impact leads to different representational geometries for the same stimuli, reducing the correlation between the resulting RDMs and highlighting how the task demands modulate the influence of optical versus dynamic information on material perception.

### Motion and shape statistics

To further examine the contribution of shape and motion cues to material perception, we conducted an exploratory analysis relating image-derived shape and motion statistics to perceptual judgments. Previous research has identified several motion and shape statistics that can predict perceived properties of materials such as viscosity in liquids or elasticity in jelly^19,22,29,36^. Here, we asked how well such relationships generalize across material categories and perceptual attributes. Overall, motion statistics showed moderate predictive power for fluidity, whereas shape statistics more strongly predicted attributes such as wobbliness and flexibility across categories. A comparison of statistical RDMs, derived from motion and shape statistics, with perceptual RDMs revealed distinct patterns. Motion statistics correlated more strongly with the rating task, whereas shape statistics correlated more strongly with the similarity judgment task. This indicates that shape information may play a more significant role when participants group materials based on overall similarity. In contrast, when explicitly rating specific material attributes, participants rely more on motion information. We suggest that this pattern arises due to the nature of the task. In the similarity judgment task, three animations are presented side by side, encouraging observers to compare stimuli holistically and group them based on readily available global structure. Under these viewing conditions, participants can align and match stimuli by their overall outline and contour configuration, so shape cues are likely to exert a stronger influence on perceptual organization. Indeed, in a large-scale study using static images of 600 material exemplars, Schmidt et al.^27^ measured perceived similarity using a triplet 2-AFC task and showed that only a limited set of 36 perceptual dimensions underlie human material perception. Many of these dimensions mapped onto shape-related cues, such as long, thin, round, and bulbous. In the rating task, by contrast, animations are presented one at a time, so observers cannot rely on side-by-side outline shape matching. Instead, they are explicitly instructed to judge specific mechanical qualities, for which dynamic cues are directly diagnostic of the rated attribute.

We also observed that the correspondence between image-derived statistics and perceptual judgments depended not only on task but also on the rendering condition. In the rating task, motion statistics RDMs correlated more strongly in the line and dot conditions than in the full condition. This indicates that when optical material cues are unavailable, observers rely more on motion-derived information to judge specific mechanical attributes. In the similarity judgment task, shape statistics RDMs showed a corresponding trend in the primary analysis, with higher correlations in the reduced conditions than in the full condition. However, when we examined distributions obtained by repeating the analysis across many randomly sampled camera angles, this trend largely attenuated (Supplementary Figure S6). However, the line condition still tended to show the highest correspondence, suggesting that when contours are the primary source of information, participants rely more on holistic grouping based on contour shape.

Importantly, the relatively lower correspondence between similarity judgments RDMs and motion statistics RDMs does not necessarily imply that contour motion is irrelevant for similarity judgments. One limitation of the current feature set is that it misses key aspects of contour dynamics as discussed in the next section. Incorporating richer spatiotemporal descriptors that explicitly track how contour geometry evolves over time may therefore increase the correspondence between image-derived statistics and perceptual organization in both rating and similarity judgments.

## Limitations and future work

Despite these systematic relationships, the findings on motion and shape statistics should be interpreted cautiously. This analysis was exploratory and was intended to characterize how relatively simple, image-derived statistics vary across material categories, rather than to identify the specific computations implemented by the visual system. The predominantly moderate effect sizes suggest that perceptual judgments may be influenced by additional factors not captured by the current feature set. One limitation is that our feature representation collapses temporal structure. We averaged the PCA scores across all frames, thereby removing information about how deformation unfolds over time. As a result, our shape statistics primarily summarize contour geometry at the level of individual frames (static shape) rather than the dynamics of shape change, and our motion statistics capture local frame-to-frame motion but, once aggregated across the sequence, do not preserve changes in motion patterns. Such deformation patterns and temporal regularities could be highly informative for material perception^20,37,41–44^.

Secondly, the motion and shape cues quantified here are not determined by material properties alone. Because our stimulus set included a diverse range of event contexts (e.g., objects falling, a jelly cube being cut, and a fabric flag flowing through the air), the resulting kinematics reflect a combination of intrinsic mechanics, external forces, and scene constraints. We therefore do not interpret these statistics as isolating pure material parameters; rather, they characterize the information available to observers under ecologically typical events. This also implies that some motion statistics may become diagnostic of category, partly because event contexts differ across categories, and that reduced displays preserve aspects of event structure that observers can use to guide material judgments. Indeed, previous research has demonstrated that the visual system effectively utilizes motion trajectories to extract information about events^45–49^. Runeson^48^ introduced the Kinematic Specification of Dynamics (KSD) framework, proposing that observers use kinematic information to infer dynamic properties of events. Building on this, Bingham et al.^45^ isolated motion information using patch-light displays of nine events spanning different dynamic categories, including rigid-body dynamics (e.g., free fall, pendulum motion, rolling ball, struck ball), biodynamics (e.g., hand-moved spring, hand-moved pendulum), hydrodynamics (e.g., stirred water, splash), and aerodynamics (e.g., falling leaves). Their findings showed that observers could successfully recognize each event based purely on motion trajectories. While they did not specifically focus on material perception, it provides valuable context for our research, reinforcing the role of dynamics in both event and material perception. Understanding how these two dimensions interact could offer deeper insights into the mechanisms underlying visual inference of physical properties. A promising direction for future research is to systematically simulate a range of events across different materials and examine how shape and motion interact to give information about the material in each context or event separately.

While the present study specifically focused on the role of motion carried by contours, dynamic dot stimuli provide an interesting case in which contour information is substantially weaker. Notably, the dynamic dot condition still showed a high correspondence with the full condition, comparable to that observed for dynamic line drawings. This pattern suggests that even when contour information is weaker, motion information can remain highly informative for material judgments. In this context, comparing static and dynamic dot displays would be especially informative, since a single static frame of a dot stimulus contains far less shape information than a static line drawing. If static-dot condition showed markedly lower similarity to full condition than both dynamic dot and static line conditions, this would further support the interpretation that judgments in the dot condition rely more strongly on motion information than on the limited shape cues available in a single frame. More broadly, this comparison would help refine our understanding of how motion and contour information jointly contribute to material perception.

## Conclusions

We conclude that contour motion in dynamic line drawings provides diagnostic information for material perception by jointly conveying contour shape and its time-varying dynamics, extending beyond what can be inferred from static contour cues alone. The strong agreement between perceptual spaces derived from attribute ratings and non-verbal similarity judgments indicates that these tasks reflect a shared underlying organization of materials. Finally, we show that even relatively simple shape and motion-derived statistics account for a meaningful portion of variance in perceptual judgments, suggesting that basic contour geometry and local motion signals contribute to perceptual material organization, while richer temporally structured dynamics likely provide additional information beyond these measures.

## Supplementary Information

Below is the link to the electronic supplementary material.

Supplementary Material 1

## Data Availability

The data and analysis scripts supporting the findings of this study are openly available in the Open Science Framework (OSF) at 10.17605/OSF.IO/CSQT5.

## Appendix

### Appendix A: rendering details

This appendix provides rendering details for each exemplar across all categories in blender.

1. Rigid-breakable: This category included four exemplars depicting hard objects composed of materials that shattered upon impact with a rigid surface. The representative frames are shown on the bottom left panel of Figure S1. Two exemplars were rigid objects thrown against an invisible wall, while the other two were rigid objects dropped from an elevation onto an invisible ground. We used the Cell Fracture add-on in Blender to shatter objects into pieces, and rigid body physics to simulate their realistic interactions upon impact. The objects thrown at the wall were identical, oval-shaped, grey objects. The impact with the wall occurred in the 7th frame in both animations, causing the object to break into 7 or 8 pieces. The difference between the two animations was that the object was rendered as hollow in one and as solid-filled in the other. The objects dropped on the ground were cubes. The impact occurred in the 12th frame in both animations, breaking the cube into 7 or 8 pieces upon impact. Consistent with the prior set, one animation consisted of the hollow cube, and the other consisted of the solid-filled cube.
2. Smoke: The stimulus set consisted of three animations (top right panel of Figure S1). Two animations show smoke: one with smoke spreading uniformly from the center, while the other displays a more dynamic dispersal pattern starting from the center. The third animation shows mist that emits from a defined source and is dispersed within an invisible box-like domain. We used Blender’s particle system in combination with fluid simulation, simulating the movement of particles through a fluid field.
3. Liquid: The stimuli for the liquid category consisted of three exemplars: two showing liquids of different viscosities constrained within a box-like domain, where the perturbed liquid flows back and forth within the boundaries of the container, and one featuring liquid droplets falling freely and splashing upon impact with the ground. The representative frames are shown in the top middle panel of Figure S1.These simulations were created using Blender’s fluid and particle systems.
4. Jelly:This category consisted of four exemplars featuring objects made of jelly, as shown in the top left panel of Figure S1. Two animations contained cube-shaped objects: one was dropped onto an invisible ground, and the other was sliced in half by an invisible knife. The third animation depicted a jelly sheet that collided with an invisible cube object when dropped onto it, bouncing up and down. The final exemplar was a potato-shaped jelly object that was also dropped onto an invisible ground. We used Blender’s particle system and the Molecular add-on to simulate jelly dynamics.
5. Fabric: This category included four animations, as shown in the bottom right panel of Figure S1. These included a soft cloth that draped over an invisible cube when dropped, a hard cloth that similarly fell over an invisible cube but retained a more rigid structure and bounced, a flag that fluttered in the wind, and a soft fabric ball filled with air that is dropped onto an invisible ground, deforming upon impact. Fabric dynamics were simulated using Blender’s cloth simulation system.

### Appendix B: calibration procedure

The calibration procedure was based on the virtual chinrest method developed by Li et al.^26^. To measure screen size, participants completed a “card task,” which involved adjusting an on-screen image of a credit card to match the physical size of an actual card placed on the screen. This adjustment was done using a slider, and the resulting card width in pixels was recorded. The procedure was repeated three times, and the average width was used to calculate the Logical Pixel Density (LPD), defined as pixels per millimeter. LPD was then used to scale the animations, ensuring a consistent physical size across different displays.

Participants were instructed to sit at an approximate viewing distance of 50 cm. However, to measure the actual distance being set, participants completed a *‘blind spot task’*. In this task, a stationary black square was presented on the right side of the screen while a red dot moved horizontally from right to left. Participants were required to cover their right eye and fixate on the stationary black square. They were instructed to press the spacebar when they perceived the dot disappearing from their sight. The distance between the center of the black square and the red dot is recorded at the moment the spacebar is pressed. The procedure was repeated 5 times, and the average of this distance was taken as the final measurement, which was then used to estimate viewing distance. Please refer to Li et al.^26^ for further details of the calibration procedure and measurements.

### Appendix C: construction of RDMs from the similarity judgment task

In a triplet 2-AFC similarity judgment task, material similarity is defined as the probability P(*i*, *j*) of participants choosing material animations (*i*) and (*j*) as belonging together, marginalized across all contexts imposed by the third animation ($\:k$).To generalize across all contexts, we needed to test each unique combination of exemplars in a triplet setting. For that, we took each of the 18 exemplars from the set of animations as a reference and paired them with every possible unique combination of exemplars from material categories distinct from the reference category. Triplets containing exemplars from the same category were excluded, as we were only interested in cross-category similarity judgments. This led to a total of 1,380 unique triplets. The whole set is then divided into four subsets with 345 triplets in one subset, which were presented in four separate runs. The order of the triplet presentations was randomized across trials within each run. For every trial, the three animations in a triplet were presented from a randomly selected camera angle, chosen from six to eight pre-rendered options. Each run consisted of four blocks, taking an average of 40 min to complete. Trial numbers and block numbers were displayed on the bottom-right and bottom-left corners of the screen, respectively, for participants’ reference.

Four runs were required to complete the entire set of 1380 unique triplets. Since it could be very tedious to complete for one participant, we provided participants with the option to choose the number of subsets they would prefer to take part in. Eighty participants completed all four runs; 3 participants completed three runs; 14 participants completed two runs; and 3 participants completed only a single run. Importantly, if a participant completed multiple runs, all were presented in the same rendering condition. Consequently, we achieved 30 repetitions for each complete set of triplets associated with each rendering condition: full, line, and line.

To estimate the perceived similarity between materials, we calculated the frequency with which a reference exemplar (*i*) was paired with each of the test exemplars (*j*) across all unique triplet combinations. This count was normalized by dividing it by the total number of unique combinations for each reference exemplar, resulting in the probability of grouping *i* and *j* together (P(*i*, *j*)), generalized across all contexts (*k*). To account for symmetry, we combined P(*i*, *j*) and P(*j*, *i*) yielding a unified probability of pairing materials *i* and *j* that ranged from 0 to 2. Finally, we subtracted these values from 2 to generate a dissimilarity matrix, which provided the required RDMs (Representational Dissimilarity Matrices) showing perceived dissimilarity between materials.
