# Supplementary material for: Perceiving material qualities from moving contours
Source: Sci Rep. 2026 Apr 14;16:12347. doi: 10.1038/s41598-026-46015-w (PMC13079875; doi:10.1038/s41598-026-46015-w)
Supplement: Supplementary file 1 — Supplementary Material 1 [file 41598_2026_46015_MOESM1_ESM.docx]

# Supplementary Figures


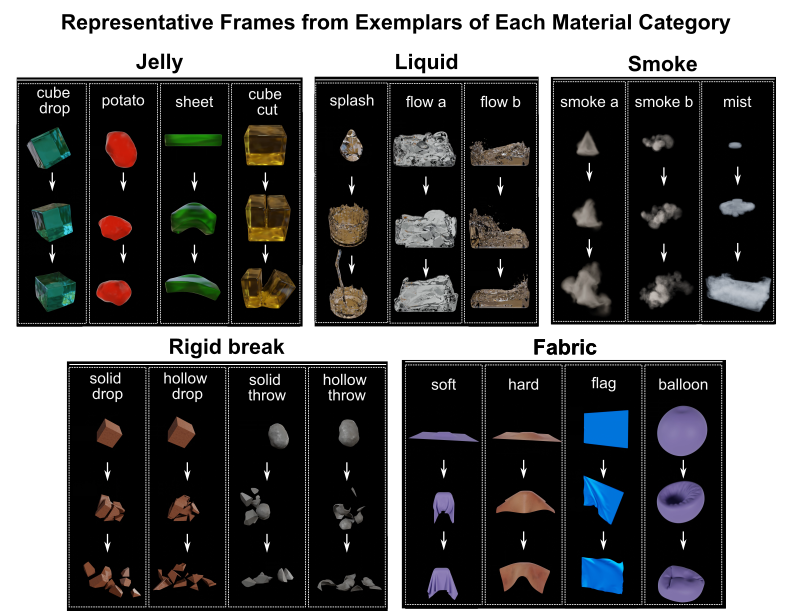


*Figure S1. The stimulus set comprises 18 exemplars, each belonging to one of the five material categories: jelly, liquid, smoke, fabric, and rigid-breakable. Three representative frames (frames 3, 15, and 40) from the total of 48 frames for each material category are shown, with arrows indicating their temporal order. While each animation was rendered from 6 to 8 camera angles, only one camera angle is displayed here.*


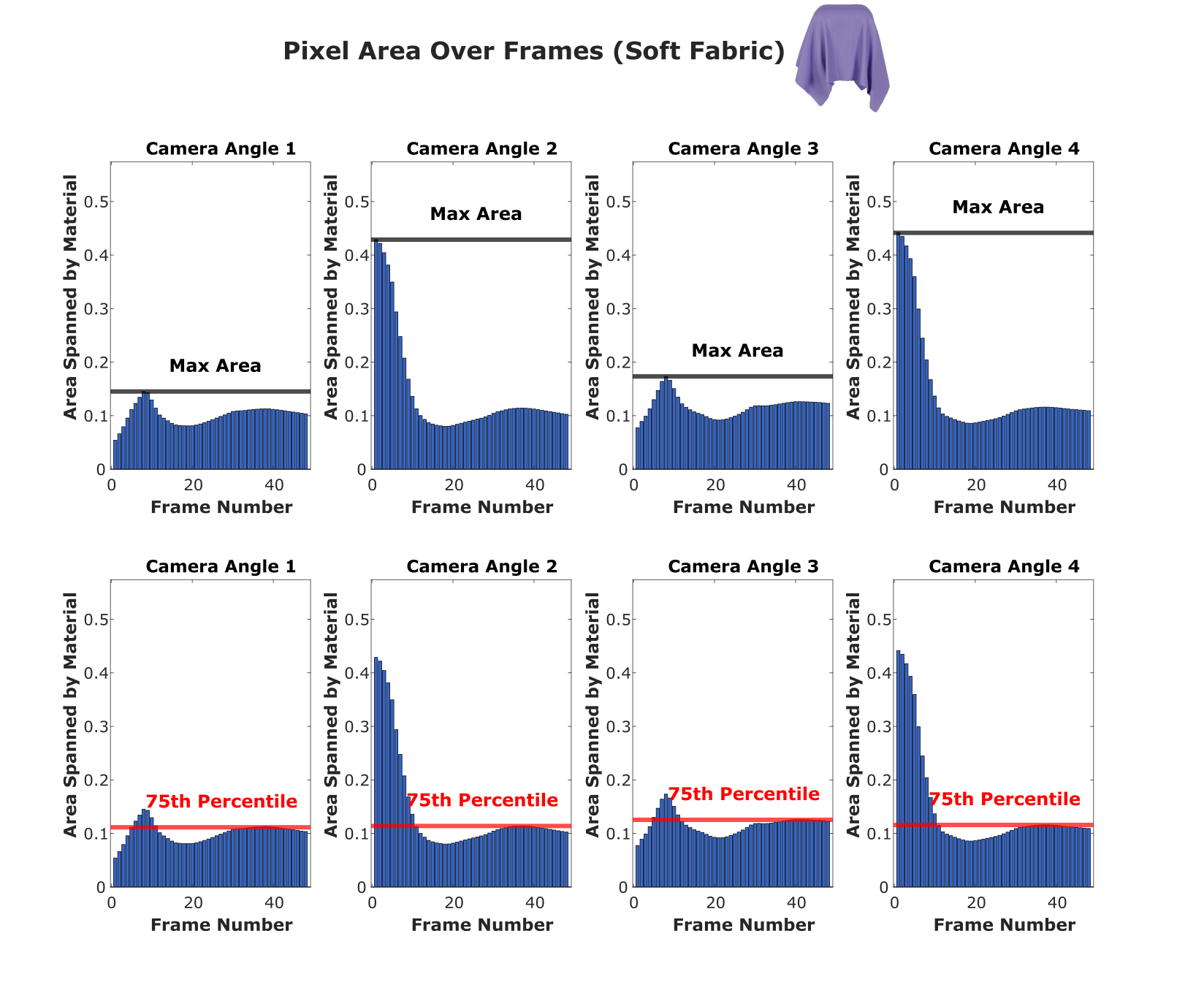


*Figure S2. The area spanned by the material across frames for one of the exemplars (soft fabric) viewed from four different camera angles. Each bar represents the proportion of pixels occupied by the material in a given frame. The top row highlights the maximum area (black line), which varies significantly across different camera angles due to differences in viewpoint and the distribution of the area per frame. Some views exhibit a relatively normal distribution of area spanned, while others are heavily skewed, leading to much higher maximum values. The bottom row highlights the 75th percentile area (red line), which remains relatively consistent across views. This choice ensured that the number of sampled vertices remained comparable, avoiding over-representing frames with extreme values and maintaining a more uniform dot density across different animations.*


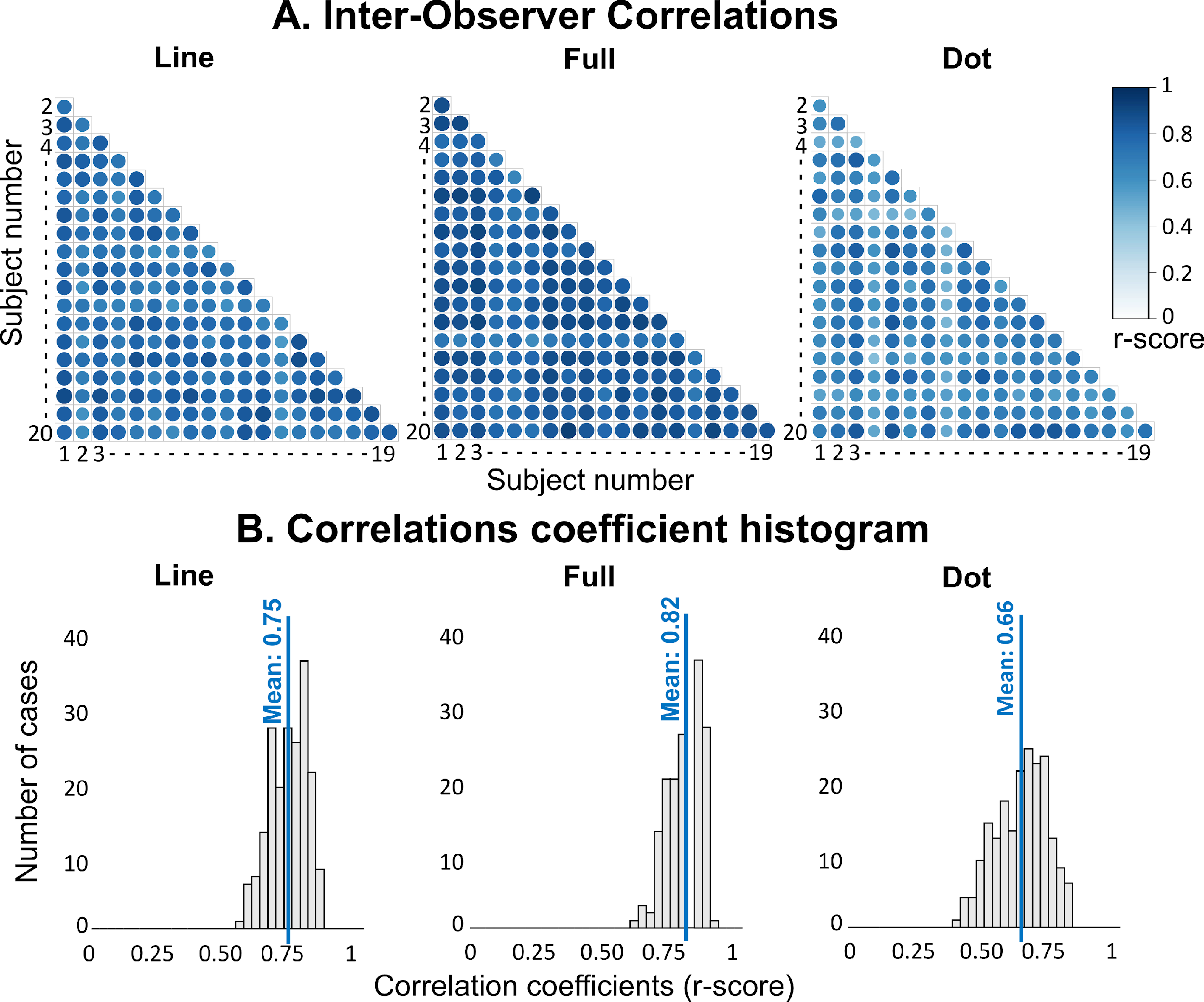


*Figure S3. Spearman correlations between participants in Experiment 1. (A) Inter-observer correlations (i.e., correlations between each participant and all other participants) for line, full, and dot conditions. Correlation coefficient values (r-scores) are represented by the color gradient shown by the bar on the right side. A lighter shade of blue indicates a lower correlation, while a darker shade indicates a higher correlation. All the correlations are significant at the level of p<0.001 and positive, ranging from 0.43 to 0.93. (B) Histogram of the correlation coefficients between all 20 participants across all stimuli and attributes tested for line, full, and dot conditions, respectively, from left to right. Mean correlations are labeled by a blue vertical line for each condition.*

*
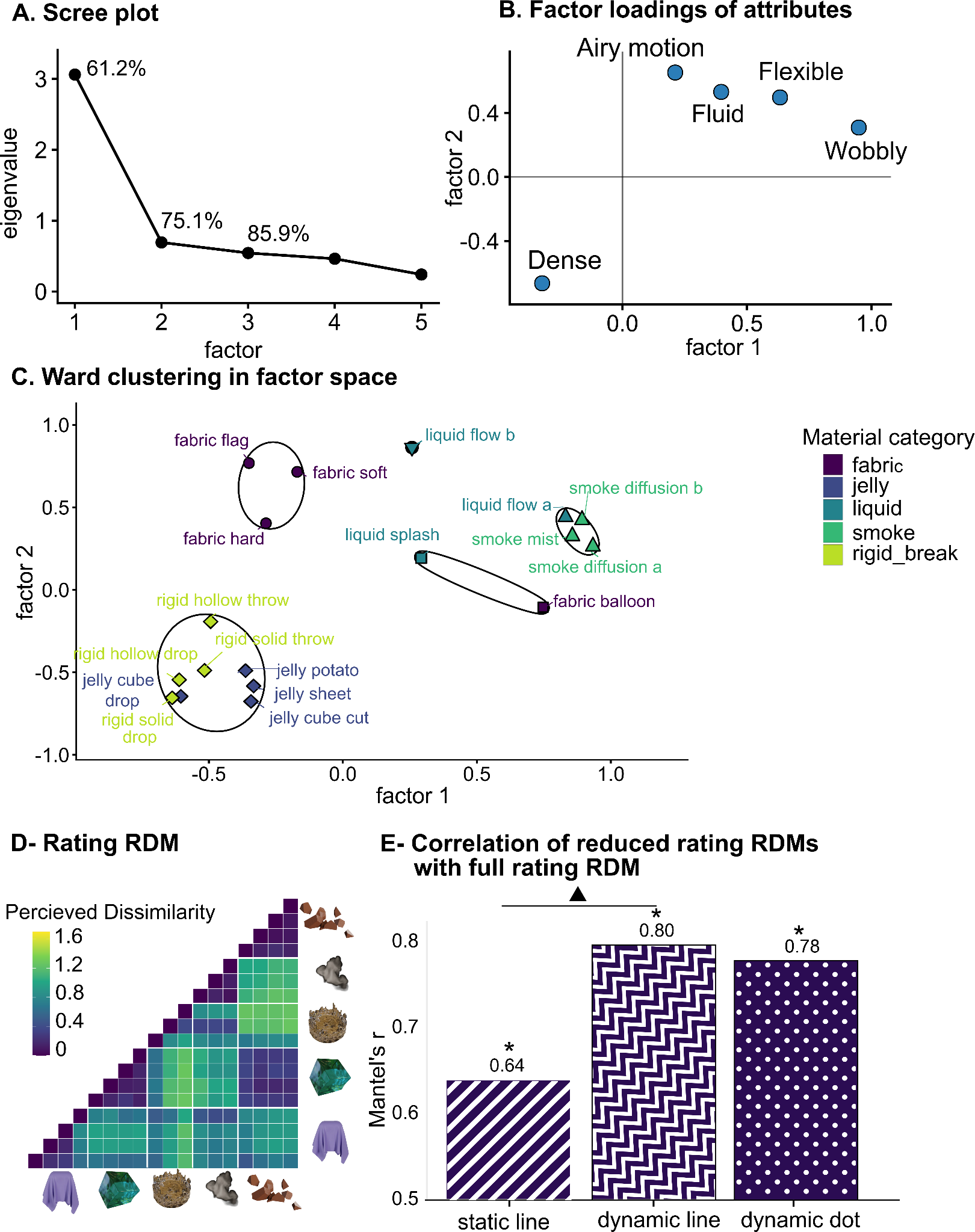
*

*Figure S4. Factor analysis, Ward clustering and RSA of attribute ratings from the static line-drawing control experiment. A) Scree plot showing variance explained by the factors in the static line condition. B) Attribute loadings for the two-factor solution in the static line condition. C) Ward hierarchical clustering based on Euclidean distances in the 2D factor-score space, cut at k=5. Different colors denote material categories, and different shapes denote cluster membership. D) Reduced RDMs correspondence with the full condition RDM for the rating task, shown as Mantel correlations (r) for static line, dynamic line, and dynamic dot conditions. Stars indicate significant correlations ( p<0.05, corrected for multiple comparisons); triangle indicates significant differences between correlation (p<0.05, corrected for multiple comparisons).*


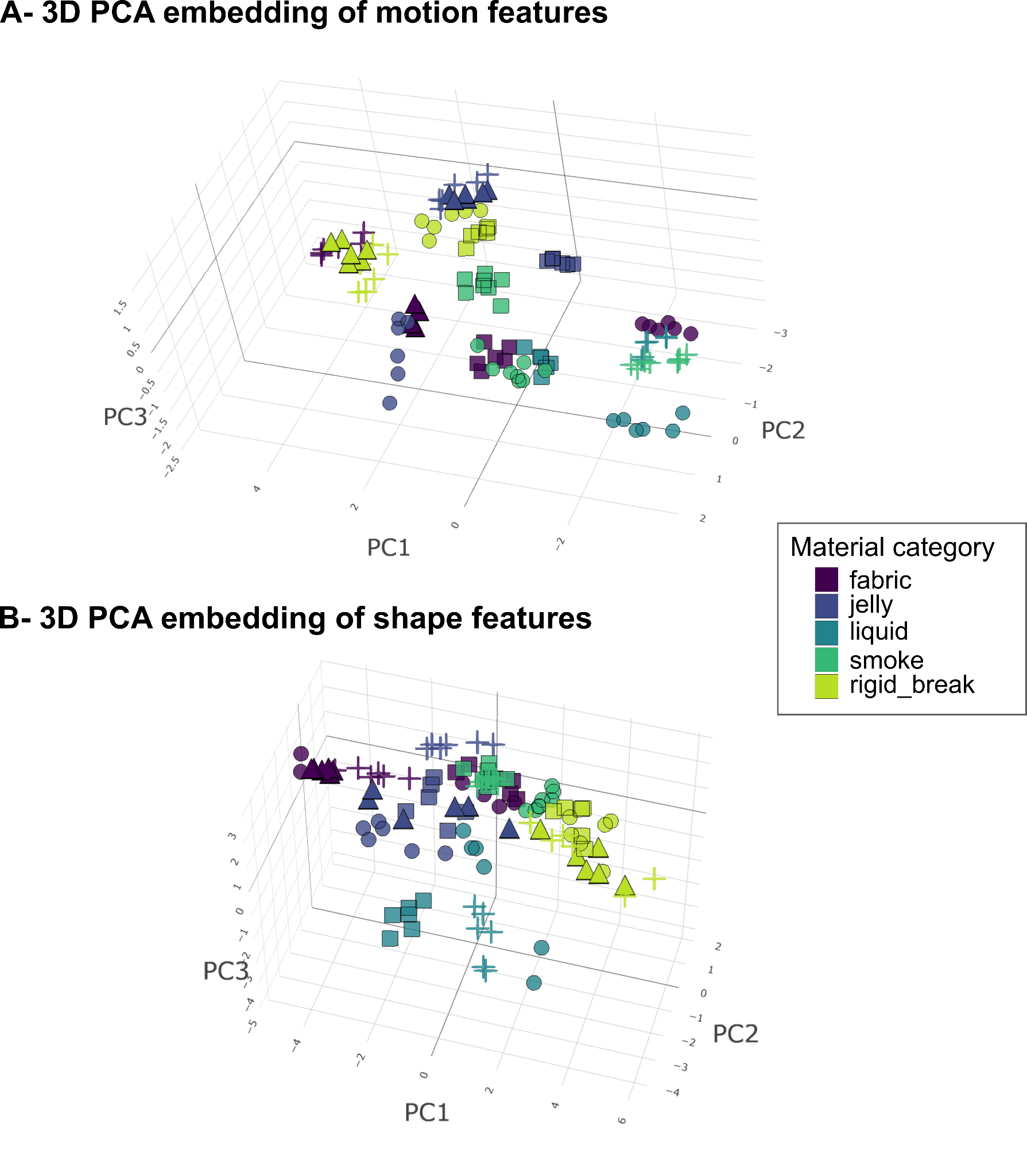


*Figure S5. PCA visualization of motion and shape statistics across materials and viewpoints. A) 3D PCA embedding motion statistics. B) 3D PCA embedding of shape statistics. Each point represents one animation from a single camera angle, positioned by the mean PC scores averaged across all 48 frames. Colors indicate material category, and marker symbols indicate exemplar identity within each category. The clustering of points with the same color and symbol reflects different camera angles of the same exemplar, showing that different camera angles occupy nearby regions in PCA space for most of exemplars.*

# *Figure S6. The complete regression and RDM analyses from Figure 8 were repeated across multiple random seeds, each time selecting a single camera angle per exemplar. Plots show the resulting distributions across camera-angle selections. (A) Adjusted R² values from regression analyses predicting the five rated material attributes from motion and shape statistics. (B) Mantel’s r values comparing motion- and shape-derived RDMs with perceptual RDMs from rating and similarity judgment tasks across the line, full, and dot conditions.*
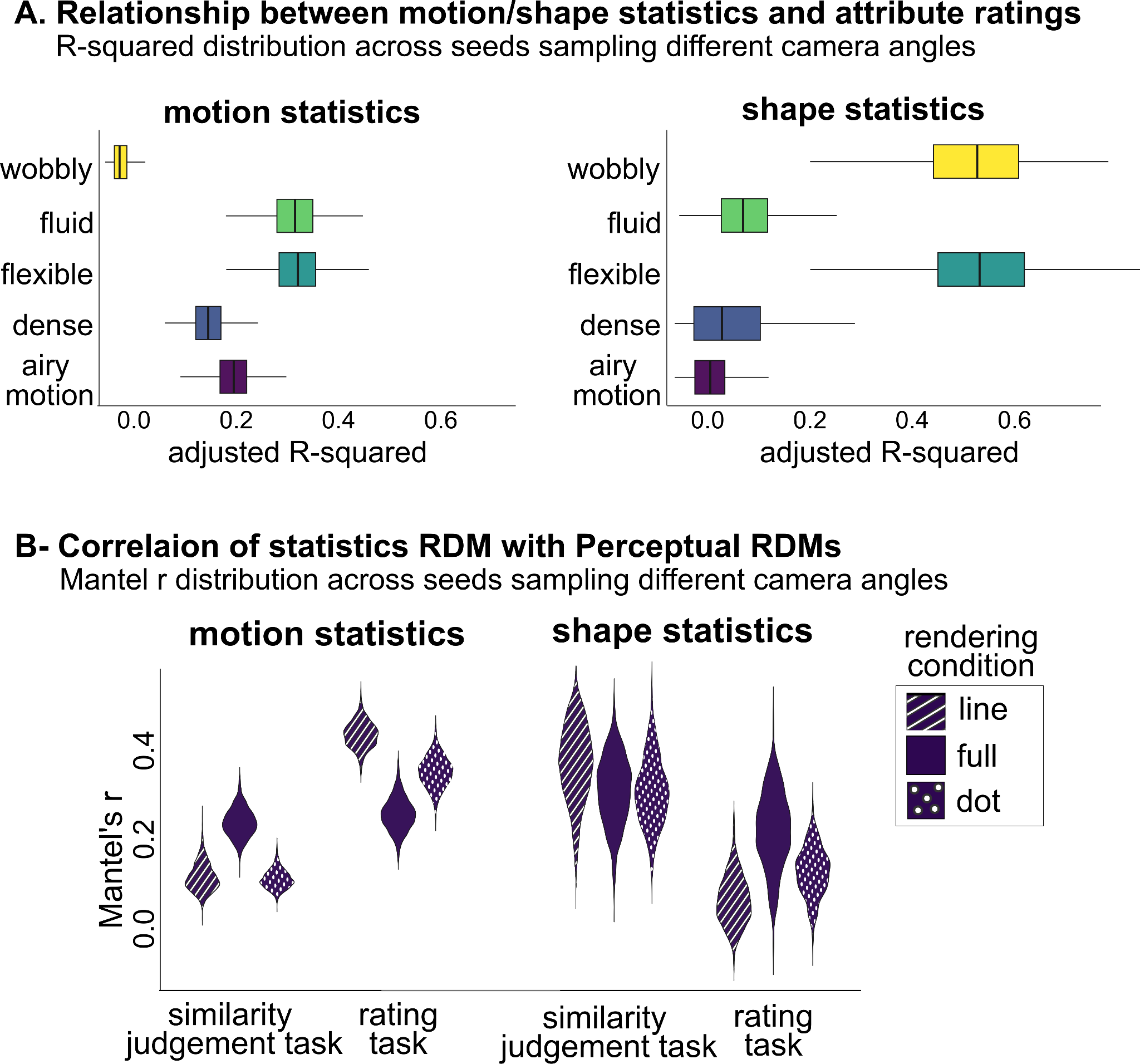


# Appendix A: Rendering Details

This appendix provides rendering details for each exemplar across all categories in Blender.

***1. Rigid-breakable:*** This category included four exemplars depicting hard objects composed of materials that shattered upon impact with a rigid surface. The representative frames are shown on the bottom left panel of Figure S1. Two exemplars were rigid objects thrown against an invisible wall, while the other two were rigid objects dropped from an elevation onto an invisible ground. We used the Cell Fracture add-on in Blender to shatter objects into pieces, and rigid body physics to simulate their realistic interactions upon impact.

The objects thrown at the wall were identical, oval-shaped, grey objects. The impact with the wall occurred in the 7th frame in both animations, causing the object to break into 7 or 8 pieces. The difference between the two animations was that the object was rendered as hollow in one and as solid-filled in the other. The objects dropped on the ground were cubes. The impact occurred in the 12th frame in both animations, breaking the cube into 7 or 8 pieces upon impact. Consistent with the prior set, one animation consisted of the hollow cube, and the other consisted of the solid-filled cube.

***2. Smoke:*** The stimulus set consisted of three animations (top right panel of Figure S1). Two animations show smoke: one with smoke spreading uniformly from the center, while the other displays a more dynamic dispersal pattern starting from the center. The third animation shows mist that emits from a defined source and is dispersed within an invisible box-like domain. We used Blender’s particle system in combination with fluid simulation, simulating the movement of particles through a fluid field.

***3. Liquid:*** The stimuli for the liquid category consisted of three exemplars: two showing liquids of different viscosities constrained within a box-like domain, where the perturbed liquid flows back and forth within the boundaries of the container, and one featuring liquid droplets falling freely and splashing upon impact with the ground. The representative frames are shown in the top middle panel of Figure S1. These simulations were created using Blender’s fluid and particle systems.

***4. Jelly:*** This category consisted of four exemplars featuring objects made of jelly, as shown in the top left panel of Figure S1. Two animations contained cube-shaped objects: one was dropped onto an invisible ground, and the other was sliced in half by an invisible knife. The third animation depicted a jelly sheet that collided with an invisible cube object when dropped onto it, bouncing up and down. The final exemplar was a potato-shaped jelly object that was also dropped onto an invisible ground. We used Blender’s particle system and the Molecular add-on to simulate jelly dynamics.

***5. Fabric:*** This category included four animations, as shown in the bottom right panel of Figure S1. These included a soft cloth that draped over an invisible cube when dropped, a hard cloth that similarly fell over an invisible cube but retained a more rigid structure and bounced, a flag that fluttered in the wind, and a soft fabric ball filled with air that is dropped onto an invisible ground, deforming upon impact. Fabric dynamics were simulated using Blender’s cloth simulation system.

# Appendix B: Calibration procedure

The calibration procedure was based on the virtual chinrest method developed by Li et al. [[26]](https://www.zotero.org/google-docs/?UWH18M). To measure screen size, participants completed a “card task,” which involved adjusting an on-screen image of a credit card to match the physical size of an actual card placed on the screen. This adjustment was done using a slider, and the resulting card width in pixels was recorded. The procedure was repeated three times, and the average width was used to calculate the Logical Pixel Density (LPD), defined as pixels per millimeter. LPD was then used to scale the animations, ensuring a consistent physical size across different displays.

Participants were instructed to sit at an approximate viewing distance of 50 cm. However, to measure the actual distance being set, participants completed a *‘blind spot task’*. In this task, a stationary black square was presented on the right side of the screen while a red dot moved horizontally from right to left. Participants were required to cover their right eye and fixate on the stationary black square. They were instructed to press the spacebar when they perceived the dot disappearing from their sight. The distance between the center of the black square and the red dot is recorded at the moment the spacebar is pressed. The procedure was repeated 5 times, and the average of this distance was taken as the final measurement, which was then used to estimate viewing distance. Please refer to Li et al. [[26]](https://www.zotero.org/google-docs/?ODxN1o) for further details of the calibration procedure and measurements.

# Appendix C: Construction of RDMs from the similarity judgment task

# In a triplet 2-AFC similarity judgment task, material similarity is defined as the probability P(*i*, *j*) of participants choosing material animations (*i*) and (*j*) as belonging together, marginalized across all contexts imposed by the third animation ($k$). To generalize across all contexts, we needed to test each unique combination of exemplars in a triplet setting. For that, we took each of the 18 exemplars from the set of animations as a reference and paired them with every possible unique combination of exemplars from material categories distinct from the reference category. Triplets containing exemplars from the same category were excluded, as we were only interested in cross-category similarity judgments. This led to a total of 1,380 unique triplets. The whole set is then divided into four subsets with 345 triplets in one subset, which were presented in four separate runs. The order of the triplet presentations was randomized across trials within each run. For every trial, the three animations in a triplet were presented from a randomly selected camera angle, chosen from six to eight pre-rendered options. Each run consisted of four blocks, taking an average of 40 minutes to complete. Trial numbers and block numbers were displayed on the bottom-right and bottom-left corners of the screen, respectively, for participants’ reference.

Four runs were required to complete the entire set of 1380 unique triplets. Since it could be very tedious to complete for one participant, we provided participants with the option to choose the number of subsets they would prefer to take part in. Eighty participants completed all four runs; 3 participants completed three runs; 14 participants completed two runs; and 3 participants completed only a single run. Importantly, if a participant completed multiple runs, all were presented in the same rendering condition. Consequently, we achieved 30 repetitions for each complete set of triplets associated with each rendering condition: full, line, and line.

To estimate the perceived similarity between materials, we calculated the frequency with which a reference exemplar (*i*) was paired with each of the test exemplars (*j*) across all unique triplet combinations. This count was normalized by dividing it by the total number of unique combinations for each reference exemplar, resulting in the probability of grouping *i* and *j* together (P(*i*, *j*)), generalized across all contexts (*k*). To account for symmetry, we combined P(*i*, *j*) and P(*j*, *i*) yielding a unified probability of pairing materials *i* and *j* that ranged from 0 to 2. Finally, we subtracted these values from 2 to generate a dissimilarity matrix, which provided the required RDMs (Representational Dissimilarity Matrices) showing perceived dissimilarity between materials.
